# The Dynamic Inflammatory Tissue Microenvironment: Signality and Disease Therapy by Biomaterials

**DOI:** 10.34133/2021/4189516

**Published:** 2021-02-03

**Authors:** Rani Mata, Yuejun Yao, Wangbei Cao, Jie Ding, Tong Zhou, Zihe Zhai, Changyou Gao

**Affiliations:** ^1^MOE Key Laboratory of Macromolecular Synthesis and Functionalization, Department of Polymer Science and Engineering, Zhejiang University, Hangzhou 310027, China; ^2^Dr. Li Dak Sum & Yip Yio Chin Center for Stem Cell and Regenerative Medicine, Zhejiang University, Hangzhou 310058, China

## Abstract

Tissue regeneration is an active multiplex process involving the dynamic inflammatory microenvironment. Under a normal physiological framework, inflammation is necessary for the systematic immunity including tissue repair and regeneration as well as returning to homeostasis. Inflammatory cellular response and metabolic mechanisms play key roles in the well-orchestrated tissue regeneration. If this response is dysregulated, it becomes chronic, which in turn causes progressive fibrosis, improper repair, and autoimmune disorders, ultimately leading to organ failure and death. Therefore, understanding of the complex inflammatory multiple player responses and their cellular metabolisms facilitates the latest insights and brings novel therapeutic methods for early diseases and modern health challenges. This review discusses the recent advances in molecular interactions of immune cells, controlled shift of pro- to anti-inflammation, reparative inflammatory metabolisms in tissue regeneration, controlling of an unfavorable microenvironment, dysregulated inflammatory diseases, and emerging therapeutic strategies including the use of biomaterials, which expand therapeutic views and briefly denote important gaps that are still prevailing.

## 1. Introduction

Tissue regeneration is a fundamental biological task essential for the survival of all organisms. Tissue repair and regeneration after mechanical injury or infection are firmly regulated complex processes involving a highly efficient inflammatory microenvironment. Inflammatory response is a body's indispensable defensive mechanism against tissue damage or pathogens [1]. After tissue damage, a quick reciprocal inflammatory response is generated in the local tissue microenvironment by the damage-associated molecular patterns (DAMPs) or pathogen-associated molecular patterns (PAMPs) via the dying and invading organisms [2, 3]. The inflammatory microenvironment facilitates various stages to restore the normal tissue framework including an early proinflammatory acute stage (initiation of recruitment of vital inflammatory cells by the innate immune response components to start the repair response), a second crucial stage (subsiding proinflammatory response by switching key proinflammatory macrophages to a repairing phenotype), and the last stage (disappearance of inflammatory cells from the injury site or elimination by apoptosis to restore tissue homeostasis). However, a sustained chronic inflammation often impairs the repair/regenerative process and forms fibrosis and scarring. It also dysregulates normal tissue functions and eventually leads to organ failure and death [4].

**Figure 1:**
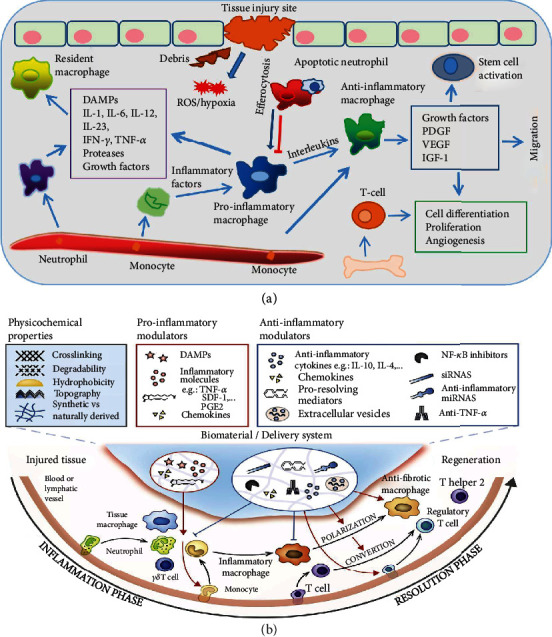
(a) Schematic illustration of the tissue microenvironment at the site of injury. Tissue injury is sensed by the resident macrophages via the released DAMPs and neutrophils that are primary infiltrating cells recruited to the damage site, which in turn recruit monocytes and macrophages. The inflammatory microenvironment is formed by the released inflammatory cytokines, growth factors, and proteases in the earlier stage. It is then shifted to the anti-inflammatory microenvironment that exploits tissue repair and homeostasis in the later stage. (b) Illustrating how the physiochemical properties of biomaterials regulate the tissue immune system. Biomaterials aid in the regulation of inflammatory cells towards the regeneration/repair phase. They are involved in the polarization of M1 inflammatory macrophages to M2 anti-inflammatory/profibrotic/proregenerative macrophages, which is a critical process for tissue regeneration. They also play a crucial role in converting T-cells into T-regulatory cells. Reprinted with permission from [21] Copyright © Elsevier 2017.

The initial acute inflammatory reaction has an intrinsic function in healing tissue injury and plays an essential role in restoring tissue homeostasis [5]. The principal goal of acute inflammation is to eliminate dead cells and pathogens at the injury site. Different types of immune cells including nonhematopoietic and hematopoietic cells collectively respond in the tissue microenvironment and together orchestrate tissue repair and regeneration [6] (Figure 1(a)). Although various cell types embrace tissue regenerative functions, the resilient macrophages play an important regulatory role. The acute inflammatory stage in skin injury encompasses stimulation of the innate immune system, resulting in initial entry of neutrophils, followed by monocytes that can be transformed to macrophages. Macrophages and other immune cells together clear the cell debris, combat against pathogens, and also organize cellular mechanisms. Such outset following the stage of new tissue formation takes place within 2-10 days after injury [7]. Multiplication and differentiation of stromal and parenchymal cells could then reconstruct tissue integrity. However, if the inflammation is not properly resolved, the granulated tissue may transform into scar tissue.

Both the migrating and local macrophages multiply and undergo remarkable phenotypic and functional modifications towards cytokines and growth factors at a local tissue microenvironment [8, 9]. Nevertheless, macrophage dysfunction could attenuate the proper tissue regeneration process and activate fibrosis formation, type I and type III collagen deposition, and myofibroblast activation. Therefore, the knowledge on how the immune cells modulate inflammation, tissue fibrosis, and neoangiogenesis would illuminate the development of promising therapies that target tissue regeneration.

A close examination on the metabolisms of immune cells over recent years has revealed a strong correlation prevailing among the metabolic state and phenotype of cells. In particular, macrophages are a notable model of this phenomenon. The M1 macrophages depend on aerobic glycolysis and fatty acid synthesis. Conversely, the M2 macrophages rely on oxidative phosphorylation (OXPHOS), tricarboxylic acid (TCA), and fatty acid oxidation (FAO) [10, 11]. Although it was believed earlier that the M1 macrophages exclusively rely on glycolysis and the M2 macrophages depend on OXPHOS as well as FAO, it has been evident that the proportion is not merely simple, and the recent evidences favor glycolysis in M2 and FAO in M1 cells [12, 13]. Therefore, the knowledge on metabolic phenotype switching provides important cues for targeting immune metabolic constituents to tune immune cell functions.

Biomaterials play a vital role in the immune modulation and macrophage polarization based on their unique physiological properties whether they stimulate or reduce inflammation. The forms of biomaterials such as micro/nanoparticles, scaffolds, and hydrogels and other properties such as crosslinking capacity, topography, and wettability as well as nature of biomaterials influence their functions [14]. The biomaterials play a significant role in regulating cell responses solely or as a part of a complicated system. The innate immune cells are the foremost cells to counter the implanted biomaterials in the vascularized tissue and consequent cell recruitment. The intensity of the acute or chronic inflammation depends on the implanted biomaterials, matrix formation, and duration. Biomaterials can be shaped into scaffolds or hydrogels for cell culture [15] and used for drug delivery [16]. They can also be easily surface modified [17] or injected [18] for different applications. The biomaterials with unique biochemical and biophysical characteristics can interact with the body and regulate the local tissue microenvironment by modulating the immune system from scarring to total regeneration. The biochemical properties of biomaterials include delivery of signaling biomolecules such as proteins and small drugs. These signaling elements released can stimulate cell-receptor proteins, which regulate the process especially protein transport, signaling, and cell morphology. For instance, biomaterials can induce angiogenesis *in vivo* within scaffolds by releasing proangiogenic growth factors [19]. Moreover, the biophysical characteristics, in particular topology, stiffness, degradation, and structure, can modulate local microenvironments via inter- and intracellular signaling [20] (Figure 1(b)). The alterations of the tissue microenvironment consist of temperature, pH, ions, and radicals. The scaffolds may direct cellular infiltration by facilitating the transport of oxygen, nutrients, and waste products and induce angiogenesis. However, when the biomaterial biophysical properties are disproportioned with tissues, it will result in low optimal healing and defective functionality of regenerated tissue. Therefore, potential biomaterials with advanced architectures (biomimicking) and surface topography (bioactive) provide well-orchestrated biomaterial-immune system interactions for optimal functionality.

This review article discusses the inflammatory microenvironment, crucial macrophage regulatory mechanisms, unfavorable inflammatory microenvironment, dysregulated inflammatory diseases, and insights into effective therapies and finally highlights recent biomaterial therapies for proper tissue repair and regeneration. Due to the increasing significance of immune-regulating biomaterials in tissue repair and regeneration and therapy of many other diseases, we wish this review can fill up the gap between immunological knowledge and biomaterials and thus promote the development of tissue microenvironment-modulating biomaterials for better medicinal applications, in particular for tissue repair and regeneration.

## 2. Inflammatory Microenvironment Signifies Tissue Repair and Regeneration

The inflammatory microenvironment at the tissue damage site is a complex interlinked framework of immune cells that play a critical role in tissue healing and homeostasis.

### 2.1. Neutrophils

Neutrophils are the primary immune cells arriving at the injury site and are critical to detecting wounds and host defence. These short-lived immune cells are briskly recruited by DAMP signals to restore barrier integrity and facilitate tissue homeostasis [22]. They can then recruit monocytes and macrophages to the inflammation site. They express a large number of chemokine receptors such as CXCR1 and CXCR2 related to the G protein-coupled receptor (GPCR) family [23]. A recent study has indicated critical roles of CXCL8 and CXCR1 or CXCR2 in fostering neutrophil stimulation and induction to the inflammation site [24].

The activated neutrophils release nuclear and granular constituents to create web-like structures of DNA, called neutrophil extracellular traps (NETs) [25, 26]. NETs comprise double-stranded DNA, histones, and granular proteins including myeloperoxidase (MPO), elastase, and cathepsin G [27, 28]. NETs are associated with pulmonary [29], inflammatory [30], and cardiovascular [31] thrombosis diseases [32]. NETs are involved in the phagocytosis of their own dying neutrophils and other cellular debris that enhance their own removal and thereby provide resolution of inflammation and tissue repair [23]. This removal process creates an anticipatory proresolution strategy characterized by the release of tissue-repairing cytokines, including interleukin 10 (IL-10) and transforming growth factor-*β* (TGF-*β*). Sofoluwe et al. have recently demonstrated that adenosine triphosphate (ATP) channel pannexin 1 (Panx1) and ATP support NET formation or NETosis, which is functionally expressed in marrow-derived neutrophils of mice (wild type) induced by the calcium ionophore A23187 or phorbol 12-myristate 13-acetate (PMA) *in vitro* [33]. They also found delayed induction of NETosis in Panx1^−/−^ mice. Therefore, the biomaterials that stimulate neutrophil apoptosis would have a great therapeutic effect on promoting tissue repair and regeneration.

However, the overall effect of neutrophil response apparently relies on the conditions including the activation of inflammatory response, tissue microenvironment, and other associated cell types. Nevertheless, various neutrophil subsets are identified based on their different functions in cancers including proinflammatory, antitumoral (N1), and anti-inflammatory protumoral (N2) phenotypes [34]. In addition, Ma et al. found neutrophil polarization during myocardial infarction (MI) and suggested that lipopolysaccharide (LPS)/interferon gamma (INF-*γ*) stimulates the polarization of blood peripheral neutrophils to proinflammatory N1, whereas IL-4 triggers the polarization of anti-inflammatory N2 similar to macrophage phenotypes [35]. The N1 neutrophil phenotype is predominant in the myocardium during MI, whereas the N2 phenotype increases over time, mitigating inflammation and promoting tissue repair. Cuartero et al. used a peroxisome proliferator-activated receptor gamma (PPAR-*γ*) agonist to polarize neutrophils to an N2 anti-inflammatory subtype, leading to beneficial results in stroke [36]. However, further investigations are required to evidently demonstrate the distinctive N2 phenotype functions. It would be quite interesting to study whether this phenotypic subset polarization takes place in various inflammatory diseases. Moreover, their phenotypic plasticity to alter from one subset to another one is a fascinating challenge because of their short life span.

### 2.2. Mast Cells

Mast cells are vital immune cells of hematopoietic lineage residing in all vascularized tissues except for the retina and nervous system [37]. They are a large source of inflammatory factors such as histamine, heparin, numerous types of cytokines, neutral proteases, and chondroitin sulfate. Migration of mast cells to target sites is achieved by the coordinated functions of chemokines, cytokines, integrins, adhesion molecules, and growth factors [38]. Although the mast cells are usually engaged in allergic responses, they are also activated during initial tissue injury and release an ample number of proinflammatory mediators including histamine and vascular endothelial growth factor (VEGF). Mast cells play an important role in recruiting inflammatory neutrophils to the injury site through protease-4 (chymase) [39]. Similar to neutrophils, the mast cells secrete numerous effector elements to recruit eosinophils and monocytes. A great number of mast cells are lethal for the tissue regeneration since they intensify acute inflammation and support scar formation in the central nervous system [40]. Moreover, they are highly present in chronic inflammatory wounds [41]. However, they also secrete anti-inflammatory molecules, indicating their bilateral and active role during the tissue repair process [40]. Nevertheless, regulation of mast cells to encourage tissue regeneration rather than scar formation is still limited.

### 2.3. Dendritic Cells

Dendritic cells are bone marrow-derived efficient antigen-presenting cells (APC) that are important modulators of innate and adaptive immunity. After tissue injury, *in situ* immature dendritic cells captivate discharged antigens and thereafter travel back to lymphoid organs through chemokine gradient, whereby initiating clonal selection and development of specific T-cells. Additionally, antigen-specific T-cells, B-cells, macrophages, natural killer cells, and eosinophils are recruited to the injured site to eliminate invasive dangers. Even though their specific roles during tissue repair and regeneration are poorly understood up to present [42], they play a crucial role during the tissue healing process [43]. Dendritic cells apparently function as immune modulators during tissue recovery via controlling macrophage homeostasis. As a subset of the dendritic cell population, the plasmacytoid dendritic cells can sense skin injury through host-generated nucleic acids identified by toll-like receptors 7 and 9 (TLR7 and TLR9) to enhance wound healing via type I INF. Wound closure of burning is considerably delayed in a dendritic cell-deficient mouse model [42]. The defective wound healing is characterized by the substantial inhibition of initial cellular proliferation, granulated tissue formation, high TGF-*β* level at the wound site, and blood vessel configuration. In a murine skeletal muscle model, dendritic cells gathering together at the injury site during regeneration undergo maturation following a target with antigen or with the exposure of LPS and migrate into a lymph node, whereby supporting antigen-specific T helper 1 (Th1) priming [44].

### 2.4. Monocytes and Macrophages

Under normal physiological conditions, macrophages reside abundantly in hematopoietic circulation and all types of tissues, which execute scavenging and maintain tissue homeostatic functions. Upon injury, huge numbers of monocytes depart from circulation and reach to the injury site. All macrophages begin their life as circulating monocytes and further reside in different tissues and habitate the local microenvironment. Both the resident and infiltrating macrophages are stimulated by the local microenvironmental cues and further mature into subpopulations characterized by the distinctive functional phenotypes. Macrophages are a great source of several cytokines, growth mediators, proteases, extracellular matrix (ECM) constituents, and soluble factors that support tissue repair, regeneration, and fibrosis [45, 46]. Macrophages are differentiated from circulatory monocytes that reach to the injury site after neutrophils in 1-3 days [47]. They reach a peak level at the injury site in around 7 days and increase to a high level up to 21 days [48]. The circulatory monocytes are classified into two different subsets based on their expression of the lymphocyte antigen 6 complex (Ly6C) marker in mice and humans. The Ly6C^+/hi^ (high) arises instantly from blood circulation, while the Ly6C^-/lo^ (low) possibly evolves from circulating Ly6C^+/hi^ monocytes [49]. They can be differentiated depending on functions. For instance, the Ly6C^+/hi^ classical monocytes respond to injury by infiltrating tissue and differentiate into macrophages during tissue regeneration. By contrast, the Ly6C^-/lo^ monocytes patrol endothelial integrity in the vascular system, but they are not differentiated into macrophages [50, 51]. After an extensive discussion on the rise of blood macrophages found at the injury site, it is now widely accepted that the infiltrating macrophages originated only from Ly6C^+/hi^ monocytes, which execute clearing damage components, wound healing, and regenerative functions [52].

However, it is still uncertain that a specific (local or recruited) macrophage can adopt a local tissue microenvironment at distinct times in response to stimulation, or they are certainly different functional monocyte subsets to regulate divergent activities. Considering these constraints of subset populations of macrophages such as M1 and M2, here we discriminate these M1/M2 subpopulations using their surface phenotype markers such as Ly6C^hi^ and Ly6C^lo^. Nevertheless, a subset of the macrophage population characterized by specific markers has divergent functions in different milieu. During the course of injury to repair, macrophages frequently shift their phenotypes between Ly6C^hi^ and Ly6C^lo^. For instance, in a CCl_4_ (carbon tetrachloride) liver injury model, the Ly6C^hi^ macrophages are proinflammatory and profibrotic, whereas the Ly6C^lo^ macrophages are anti-inflammatory and antifibrotic. Moreover, in various tissues including skeletal muscle and renal tissue, the Ly6C^hi^ macrophage promotes inflammation and the Ly6C^lo^ macrophage stimulates fibrogenesis [53, 54].

A proinflammatory Ly6C^hi^ CX3CR1^lo^ macrophage population derived from monocytes acquires homing at the injury site in a chemokine ligand 2- (CCL2-) dependent fashion. The reparative Ly6C^lo^ CX3CR1^hi^ subpopulation moves along the way led by cluster of differentiation 73 (CD73), vascular cell adhesion protein 1 (VCAM-1), very late antigen-4 (VLA-4), endothelial-cell-surface enzymes (of leukocyte extravasation), and adhesion proteins [55]. Lorchner et al. identified that regenerative islet-derived 3*β* (Reg3*β*) is an important modulator of macrophage to cardiac tissue after injury [56]. The Reg3*β* facilitates recruitment of the repair macrophage population that assists neutrophil removal, or else it would stimulate matrix degeneration and delay collagen accumulation and myocyte rupture.

In many tissues, an individual subset of monocytes can function as proinflammatory and proreparatory cells, indicating that *in situ* modulation rather than provision of the proreparative LyC6^−^ population is crucial in many contexts. For instance, the protein activin-A that guides differentiation of oligodendrocytes during remyelination of the central nervous system (CNS) is a critical macrophage-derived proreparative factor, and inhibition of M2-derived activin-A protein blocks the differentiation of oligodendrocytes during remyelination in culture of cerebellar slices [57]. In response to the stimuli, macrophages have a broad range of activities particularly in the transition of innate immune response through the mediation of various factors. Indeed, some of these functions are distinct.

The opposing roles of macrophages, for example, pro- and anti-inflammatory nature, rely on stimuli that trigger to procure a divergent phenotype. Despite the fact that the microenvironmental factors and phenotypes are divergent, they are broadly classified into two phenotypes depending on the type of T helper cell type 1 or 2 (Th1/Th2) cell polarization [58]. The IFN-*γ* and tumor necrosis factor alpha (TNF-*α*) excreted by Th1 cells stimulate macrophages to a classically activated M1 phenotype, and the Th2 cytokines including IL-4 and IL-10 can inhibit stimulation of macrophages, which are identified as an anti-inflammatory M2 phenotype [59]. The M1 macrophages primarily assist in phagocytosis, as well as in IL-12-associated Th1 effects, whereas the anti-inflammatory M2 phenotype performs Th2 responses and serves in the later phases of tissue repair. By means of the microenvironmental factors, the M2 phenotype is additionally subclassified into M2a, M2b, M2c, and M2d subtypes. The M2a subtype is notably a profibrotic subset activated via IL-4 and IL-13 [60]. The M2b subtype is triggered by the combined exposure with immune complexes as well as toll-like receptors (TLR)/IL-1 antagonist [61]. The M2c subtype is triggered by the IL-10 and TGF-*β*/glucocorticoids that suppress inflammation and promote neovascularization [60]. The M2d macrophages are considered tumor-associated macrophages (TAMs) due to their involvement in the tumor growth, angiogenesis, and metastasis [62]. They are activated by IL-6, TLR, and A2 adenosine receptor (A2R) agonists and produce higher levels of IL-10, TGF-*β*, and VEGF but minimal levels of IL-12, TNF-*α*, and IL-1*β*. The classification paradigm of the M1/M2 axis encounters several controversies because it is based on the *in vitro* construction on stimulating macrophages in culture with a specified set of factors and hence may miss the *in vivo* setting of tissue-specific gene expression programs. For example, *in vitro* stimulation of monocytes using macrophage colony-stimulating factor (M-CSF) or granulocyte macrophage colony-stimulating factor (GM-CSF) indicates deviation from the M1/M2 concept, because free fatty acids and high-density lipoproteins are within such stimuli, which is specially applicable to the cardiovascular investigators. Therefore, the classification needs more keen observation on natural habitats over simply naming according to functions because they may be changed at different environments [63].

Overall, the earlier investigations with various experimental schemes in different organ systems properly highlight the unique and opposing role of inflammatory monocytes and tissue-resident macrophages in the tissue repair and regenerative process [63]. The critical decisive roles of macrophages from proinflammatory to anti-inflammatory subsets during regenerative response are discussed extensively in the following section.

### 2.5. T-Cells

Over the past decade, innate immunity including the macrophage phenotype polarization has been addressed as a critical player in the tissue regeneration process. Nevertheless, recent studies indicate that adaptive immunity including T-cells also performs a central role. The T-cell types and subpopulations accumulating at the injury site greatly vary in different tissues. For example, in bone, both the Th1 CD4^+^ and cytotoxic CD8^+^ subpopulations restrict regeneration. T-cells suppress major histocompatibility complex- (MHC-) derived bone generation in a mouse through regulating IFN-*γ* and TNF-*α* [64]. The investigation in humans shows that the excretion of IFN-*γ* and TNF-*α* results in delayed osteogenesis and fracture healing [63].

Although the regulatory T-cells (T-regs) have an indirect role in regulating inflammation induced by injury, they also act directly through amphiregulin, which is a molecule produced by different immune cells and plays a central role in organ development and supports tissue regeneration in the context of inflammation [65]. The highly accepted reliable specific T-reg marker is forkhead box P3 (FOXP3), which is vital for the maturation and function of T-regs. An excessive inflammation after tissue injury causes defective tissue healing and remodeling. T-regs from various tissues are recruited to the injury site to promote inflammation resolution and control immunity [66], where they can indirectly modulate regeneration by regulating neutrophils, stimulating macrophage polarization, and controlling T helper cells [67, 68]. Furthermore, T-regs can directly support regeneration via stimulating local progenitor cells [69]. A recent *in vitro* study has highlighted that the activated T-regs facilitate neutrophils to excrete anti-inflammatory mediators such as heme oxygenase-1, indoleamine 2, 3-dioxygenase (IDO), IL-10, and TGF-*β* through the preinhibition of IL-6 production, suggesting that T-regs can modulate inflammation via inhibiting neutrophil function [70]. Moreover, T-regs can control infiltration of neutrophils to the damage site. For example, removal of T-regs can cause the increase in neutrophil infiltration upon cardiac injury and consequently results in a defective healing process [66]. Many studies have evidenced that CD4^+^ T-regs are essential for the repair and regeneration of various tissues such as skeletal muscle, lung, myocardium, bone, kidney, and skin [71–75].

### 2.6. Pro- to Anti-Inflammatory Macrophage Modulation for Tissue Regeneration

The proinflammatory macrophages known as M1 macrophages can be polarized into anti-inflammatory macrophages or alternatively activated M2 macrophages. This M1-M2 macrophage modulation is crucial for scavenging inflammation and promoting tissue repair. In case if the early inflammation is not regulated, excessive inflammation can impair the tissue repair process. Alternatively, an immature early anti-inflammation process could interrupt the tissue repair and regeneration process. IFN-*γ* is an important cytokine involved in the activation of M1 macrophages. The IL-4 and IL-13 are typical stimulating factors for inducing the M2 phenotype. Alternatively, the anti-inflammatory IL-10, intermediates of glucose and lipid metabolism, prostaglandins, and glucocorticoids, may also induce the M2-like phenotype. The anti-inflammatory IL-10 plays an essential role in the polarization of macrophages from proinflammation to anti-inflammation, which boosts muscle regeneration [76]. Nevertheless, IL-4 is often transmitted as a protein to trigger M(IL-4)-like macrophage transition. For instance, in an *in vivo* rat model with peripheral nerve damage, IL-4 is supplied through the injectable agarose hydrogels to enhance the number of M(IL-4) macrophages [77].

Moreover, the controlled delivery of IL-4 supports the repair of peripheral nerve via M(IL-4) macrophages. Overall, the release of IL-4 possibly improves tissue repair and regeneration in various contexts by inducing M(IL-4) macrophages. However, the IL-4 effect on the T-regs still has to be explored. The T-reg accumulation at the injury site suggests the modulation of macrophage phenotypes, supporting their contribution to the transition of macrophages through IL-10 and other regulators. In a previous study, early reduction in T-regs upon injury fosters NK cells and effector T cells to produce IFN-*γ*, resulting in increased inflammatory stimulation of macrophages [78]. Similar to anti-inflammatory M(IL-4) macrophages, mesenchymal stem cells also play a regulatory role by switching resident macrophages from a proinflammatory M(IFN-*γ*) phenotype to a tissue-regenerative phenotype [79]. However, the M(IL-4) cells simultaneously develop an anti-inflammatory microenvironment that facilitates persistence and growth of both MHCs and progenitor cells at the injury site, indicating the existence of a reciprocal helpful feedback relationship among anti-inflammatory macrophages and pluripotent stem cell subsets that promotes tissue regeneration [80, 81].

Indeed, impairment in the polarization of M1 to M2 macrophages has been involved in the pathogenic chronic wounds. In the diabetic wound repair process, activation of PPAR-*γ* is critical for the phenotype transition of macrophages defected by the steady manifestation of IL-1*β* in mouse and human wounds. This causes impaired macrophage shifting from M1 to M2, leading to a delayed wound healing process [82]. The later stage of the wound healing process involves removal of neutrophils, because the overtime accumulated neutrophils produce proteases. The overproduced proteases can deteriorate the complement system, ECM, clotting factors, immunoglobulins, and cytokines, which are essential for tissue repair and regeneration. Moreover, they also produce reactive oxygen species (ROS) that further impair and delay the tissue healing process. Hence, the clearing of neutrophils is demanded to advance the repair process into a proliferative stage. The macrophages play a major role in the removal of neutrophils through apoptosis and then by phagocytosis referred to as efferocytosis [83, 84]. It is noteworthy that the efferocytosis of neutrophils by macrophages is a critical element to stimulate modulation from a pro- to anti-inflammatory phenotype [85, 86]. For instance, lung macrophages induce the engulfment of apoptotic bodies by the epithelial cells of the lung airway via insulin-like growth factor 1 (IGF-1) [87]. A recent study shows that the developmental endothelial locus-1- (DEL-1-) secreted protein inhibits adhesion of leukocytes and endothelial cells and initiation of inflammation and thus functions in resolving inflammation. The proresolving action of DEL-1 can be attributed to the efferocytosis via reprogramming liver X receptor- (LXR-) dependent macrophages to a proresolving phenotype with the help of specific proresolving mediators [88].

### 2.7. Metabolisms Linked to Proinflammatory and Prorepair Functions of Immune Cells

Macrophages are familiar in embracing the challenging circumstances to fight infection and promote repair, but their precise energetic needs in this setting are not fully understood. Nevertheless, the macrophages receive their bioenergy via glycolysis or oxidative metabolism, which can cause various phenotypes. Mitochondria are the powerhouses of immune cells [89]. Metabolic intermediates not only are a source of energy but also are directly involved in metabolic switching of phenotypes of different immune cells including macrophages, neutrophils, dendritic cells, T-cells, T-regs, and memory T-cells (Table 1).

#### 2.7.1. Glycolysis

The M1 classically activated macrophages are major regulators of primary defence infections and gain energy via glycolysis [90], whereas the alternatively activated M2 macrophages obtain energy from oxidative metabolism to boost their long-term activities [93]. Indeed, the activation of M1 macrophages stimulates the glycolysis pathway that entails glucose uptake and conversion of pyruvate to lactate. Meanwhile, ROS are produced by impairing the respiratory chain functions.

The hypoxia-inducible factor 1-alpha (HIF-1*α*) is a transcription factor that regulates transcriptional programming of glycolytic metabolism and production of proinflammatory cytokines for activating M1 macrophages [102]. Moreover, the accumulated ROS by the impaired TCA cycle following inflammatory circumstances stabilize HIF-1*α* and uphold the M1 phenotype [103]. Similarly, succinate oxidation by succinate dehydrogenase (SDH) sustains the LPS-mediated proinflammatory phenotype and triggers IL-1*β* production through HIF-1*α*, which can be shut down by inhibiting glycolysis using 2-deoxyglucose (2-DG) [103, 104]. This response illustrates that the macrophage metabolism not only provides an energy source but also is involved directly in the transcription modulation of immune function. Another metabolite itaconate of LPS-stimulated macrophages suppresses inflammation by blocking SDH [105]. However, in HIF-1*α*-deficient cells, the LPS may be involved in stimulation of nuclear factor kappa-light-chain-enhancer of activated B (NF-*κ*B) via a ubiquitous phosphofructokinase-2 (uPFK2) regulatory enzyme of glycolysis [106]. Deactivation of HIF-1*α* in myeloid cells substantially reduces the manifestation of glucose transporter 1 (GLUT1) and commencement of glycolytic cascade. Another glycolytic regulatory enzyme pyruvate dehydrogenase kinase 1 (PDK1) is also essential for the HIF-1*α* in M1 macrophages [107]. Glycolytic switch takes place before the oxygen level of mitochondria inhibits respiration, indicating preemptive adaptation that is greatly related to the physiological event, since inflammatory cells move down to oxygen gradient. However, with the failure of HIF-1*α*, PDK1 inhibition causes defective macrophage migration. Additionally, the shortage of myeloid cell prolyl-hydroxylase 2 (PHD2) promotes the anaerobic glycolysis in a HIF-1*α*- and PDK1-dependent manner [108]. In LPS-stimulated cells, glycolysis is ameliorated through the induction of an enzyme pyruvate kinase isoenzyme M2 (PKM2) [109]. Alternately, PKM2 translocates into the nucleus, wherein it interacts with HIF-1*α* and facilitates the expression of HIF-1*α*-related genes that translate into glycolytic enzymes as well as inflammatory factors including IL-1*β* [109]. It is exciting to observe that the PKM2 nuclear tetrameric form can rewrite gene expression profiling of macrophages to obtain ones more like the M2 phenotype. This reprogramming suggests that the regulation of HIF-1*α* indeed can shift proinflammatory M1 to a proreparative M2 phenotype. Another fascinating glycolytic enzyme glyceraldehyde 3-phosphate dehydrogenase (GAPDH) induces glycolysis in the activated immune cells by binding to messenger RNA (mRNA) that encodes IFN-*γ* [110].

**Figure 2:**
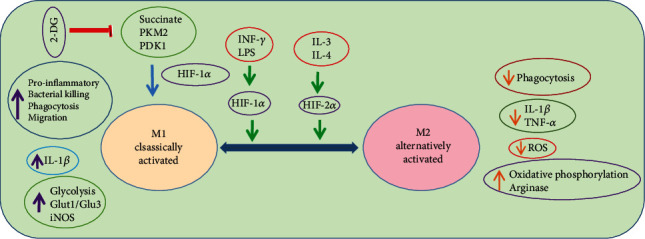
Regulatory functions of HIF-1*α* and HIF-2*α* in M1 and M2 macrophages. HIF-1*α* modulates the glycolytic pathway of M1 macrophages and inflammatory cytokine production. HIF-2*α* regulates the alternatively activated M2 macrophages through activating arginase and oxidative phosphorylation.

Although the effective role of HIF-2*α* in stimulating alternatively activated M2 phenotype is promising, the mechanism remains unclear. HIF-2*α* perhaps regulates the M2 transcription factor Arg1. However, there is discrepancy over the role of HIF-2*α* because it can also stimulate the production of IL-1*β* related to the M1 phenotype [103]. These studies demonstrate that both the isoforms of HIF-1*α* and HIF-2*α* apparently have redundant and overlaying actions (Figure 2). Even if one is silenced, the other could not be capable of compensating. Therefore, there are still major loopholes in our knowledge on the various functional roles of isoforms of HIF-1*α* and HIF-2*α*.

The prolyl-hydroxylases (PHD) 1-3 are 2-oxoglutarate-dependent dioxygenase (2-OGDD) enzymes that play a pivotal role in the regulation of HIF-1*α*. The HIF-1*α* proline moieties positioned on the oxygen-dependent degradation domain are hydroxylated by the actions of PHDs. Following a low oxygen condition or at declined levels of alpha-keto glutarate (*α*-KG) or Fe^2+^, the PHD actions are altered, leading to HIF-1*α* or HIF-2*α* accumulation and translocation to the nucleus wherein the expression of genes related to metabolism of immune cells is regulated [111]. The iron regulatory proteins (IRPs), namely, IRP2, control the metabolic processes by switching glycolysis to OXPHOS in mouse embryonic fibroblasts via HIF-1*α* and HIF-2*α* [112].

#### 2.7.2. Pentose Phosphate Pathway

Alternatively, the pentose phosphate pathway (PPP) is also increased in the LPS-activated macrophages and is essential for the production of nicotinamide adenine dinucleotide phosphate (NADPH) required for NADPH oxidase that is crucial for the ROS production and nitric oxide (NO) synthesis [103]. The critical regulatory enzyme of this pathway is a carbohydrate kinase-like (CARKL) protein, also known as sedoheptulose kinase, which controls the flux of PPP and is greatly expressed in M2 macrophages [113]. If the CARKL protein is suppressed, macrophages become more like M1, suggesting the PPP role in the modulation of M1 macrophages. However, it is still unclear why the M1 macrophages favor PPP, although the cells exhibit low proliferation capability. In contrast, the M2 macrophages obtain their energy from oxidative and fatty acid oxidative metabolisms.

#### 2.7.3. Oxidative Phosphorylation (OXPHOS)

The OXPHOS is involved in the electron gradient that creates a proton motif force essential for the ATP generation through the mitochondrial complexes. The M1 macrophages activated by LPS are found to have a dysregulated Krebs cycle and OXPHOS pathways [114]. These metabolic transitions support brisk ATP generation to uphold their phagocytic actions and supply intermediates to sustain PPP in M1 macrophages. An overexpression of GLUT-1 in M1 macrophages leads to the increased uptake of glucose, which in turn increases the numbers of PPP intermediates and reduces OXPHOS [115]. In contrast, M2 and T-regs favor to procure anti-inflammation phenotype functions.

The monocytes utilize OXPHOS and glycolysis to fulfill energy demands that differ based on the immune response whether anti-inflammatory or resolution phase. The thioredoxin 1 (Trx1) reduction of Cys in HIF-1*α* and Fe-S clusters is expected to increase the efficiency of oxidative phosphorylation and inhibit glycolytic gene expression due to HIF-1*α* degradation. Upon stimulation, the M2 macrophages induce electron transport chain components to perform OXPHOS and drive pyruvate into the Krebs cycle. The M2 phenotype can be inhibited by blocking OXPHOS, thereby driving the M1 macrophage phenotype. Further constraining of oxidative metabolism in the M1 macrophage reinforces the M2 phenotype [106]. Macrophages activated upon exposure with IL-4 can stimulate the transcription factor signal transducer and activator of transcription 6 (STAT6), which further induces protein PPAR-*γ*-coactivator-1*β* (PGC-1*β*) that is responsible for the induction of mitochondrial respiration and biogenesis. Along with transcription factors, nuclear respiratory factor 1 (NRF-1) and estrogen-related receptor *α* (ERR*α*) also can drive the generation of important mitochondrial complexes including cytochrome c and ATP synthase [116]. Indeed, the knockdown of PGC-1*β* ruins the metabolic M2 macrophage profile and also their functions [117]. The PPARs especially PPAR-*γ* and PPAR-*δ* play a critical role in sustaining the M2 phenotype via macrophage galactose-type C-type lectin 1 (MGL-1) and *β*-oxidation of fatty acids, respectively [118, 119]. Although the metabolic variations in macrophages are largely agreed, the switches that orchestrate distinct phenotypes at the molecular level remain greatly unclear.

#### 2.7.4. TCA or Citric Acid or Krebs Pathway

**Figure 3:**
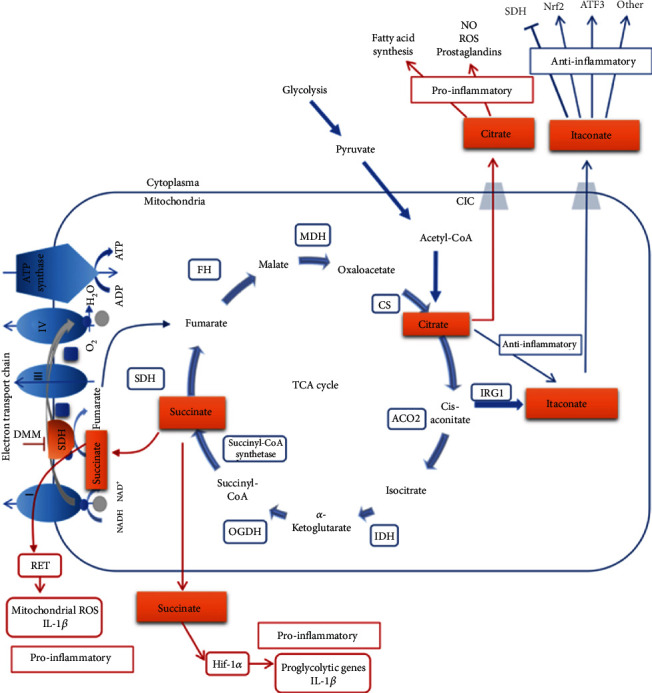
Overview of the principal metabolic regulation of TCA, glycolysis, electron transport chain, and fatty acid synthesis in macrophages involved in switching macrophage phenotypes. Glycolysis is primarily involved in the activation of M1 that further secretes proinflammatory cytokines. The TCA cycle mainly supports the stimulation of M2 macrophages, consequently inducing secretion of prorepair cytokines. The important metabolites or enzymes involved in the phenotype switching are highlighted in orange color. Reprinted with permission from [134] Copyright © 2020 Springer Nature.

TCA is a general aerobic pathway of mitochondrial respiration in cells. TCA or the Krebs pathway functions in providing energy as well as metabolic intermediates including citrate, *α*-KG, succinate, fumarate, and malate that regulate many cellular responses via several signaling pathways (Figure 3). An impaired Krebs cycle has been found in LPS-activated M1 macrophages [114]. The important Krebs cycle intermediate, namely, citrate, plays a key role in the immuno-metabolic modulation of immune cells. The higher isocitrate/*α*-KG ratio induces downregulation of the transcriptional factor Idh1 observed in M1 classical macrophages and dendritic cells [120, 121]. The impaired Krebs cycle and enhanced glycolysis flux drive pyruvate, which enters into the Krebs cycle but is unable to pass citrate/isocitrate. An elevated level of citrate is discovered in mouse macrophages stimulated by LPS and human macrophages triggered by TNF-*α* or IFN-*γ* [122]. This is in turn associated with the upregulation of mitochondrial citrate carrier (CIC) and ATP-citrate lyase (ACLY) via NF-*κ*B in LPS- or TNF-*α*-induced immune cells. On the other hand, IFN-*γ*-stimulated cells induce CIC and ACLY through STAT1 [123]. The breakdown of the Krebs cycle and the accumulation of citrate are related to the generation of crucial proinflammatory mediators in humans including prostaglandin E2 (PGE2), NO, and ROS [122, 123]. Intriguingly, the Akt-mammalian target of rapamycin 1 (mTORC1) signaling pathway also regulates the function of ACLY and protein levels in IL-4-stimulated macrophages [124]. Alternatively, the accumulated citrate formed by the key aspect of IDH1 can be utilized to stimulate fatty acids and acetylation of histone, and the other fate is the generation of itaconate [125].

Itaconate has recently come under spotlight in the area of immune cell metabolisms because of its efficient anti-inflammatory regulator capacity. Upon LPS exposure, the M1 macrophages largely produce itaconate metabolite in the Krebs cycle from citrate [105]. The mitochondrial enzyme aconitase 2 (ACO2) acts on citrate and produces *cis*-aconitate [126], which is further decarboxlylated to drive itaconate by the enzyme cis-aconitate decarboxylase, referred to as immune-responsive gene 1 (*IRG1*). Treatment with dimethyl itaconate (DMI), a permeable analog of itaconate in murine bone marrow-derived macrophages (BMDMs) stimulated with LPS, can downregulate the expression of various proinflammatory genes along with inducible nitric oxide synthase (iNOS) and suppress the production of IL-6, IL-18, IL-1*β*, ROS, and NO [105]. It has been shown that itaconate can also prevent the functions of SDH that is mitochondrial component complex II of the electron transport chain (ETC) [127] and further inhibit ROS generation by reverse electron transport (RET) [128]. Although the usage of DMI provides insights into the regulatory function of itaconate, there are still some questions that need to be answered, for example, how the gene silencing of *IRG1* and prominent amount of *IRG1* mRNA as well as itaconate synthesis are enhanced in immune cells. Itaconate also triggers electrophilic stress and binds with glutathione and consequently stimulates both nuclear factor erythroid 2-related factor 2- (Nrf2-) dependent [129] and independent reactions. Bambouskova et al. found that this selective electrophilic stress regulates secondary transcriptional response rather than primary transcriptional response to activate toll-like receptor via inhibiting inhibitor of kappa B-*ζ* (I*κ*B-*ζ*) protein induction that is independent of Nrf2 but dependent on a key mediator activating transcription factor 3 (ATF3) [130].

The key enzyme of the Krebs cycle *α*-KG plays critical functional roles in promoting an anti-inflammatory M2 macrophage phenotype while suppressing proinflammatory responses [131]. The M2 macrophages activate the expression of an array of scavenging receptors including mannose receptors that function in identification and phagocytosis of apoptotic cells. In IL-4-activated M2 macrophages, the TCA pathway imparts in the production of uridine 5′-diphospho-N-acetylglucosamine (UDP-GlcNAc), an essential intermediate necessary for the glycosylation of the mannose receptor [120]. In contrast, chemical inhibition of the OXPHOS pathway enzyme ATP synthase in IL-4-activated M2 macrophages reduces the functional expression of M2 genes such as Arg1, C-type mannose receptor 1 (Mrc1), and markers CD206 and arginase-1 [132]. The main cause of the increased glutaminolysis in the proinflammatory IL-4-activated macrophages is the high level of *α*-KG, resulting in the promotion of the anti-inflammatory M2 phenotype through regulating the histone demethylase Jumonji domain-containing histone demethylase 3 (Jmjd3) or T5-methylcytosine hydroxylases (TET). In contrast, in LPS-activated M1 macrophages, the low level of *α*-KG reduces proinflammatory functions. The *α*-KG represses the inhibitor of nuclear factor kappa-B kinase (IKK*β*) activation needed for the proinflammatory functions driven through the NF-*κ*B pathway depending on PHD activity. These results emphasize the targeting schemes involved in *α*-KG production, suggesting a fascinating therapeutic possibility in impaired macrophage-associated diseases.

The alternatively activated M2 macrophages also induce arginine metabolism through arginase-1 (Arg-1), resulting in the generation of ornithine, urea, and polyamines that are crucial for the wound healing functions of M2 macrophages [133]. Recently, a key protein TNF-*α*-induced protein 8-like 2 also known as TIPE2 has been reported, which can trigger an M2 phenotype through inducing arginine metabolism. Fascinatingly, TIPE2 strives these actions upon long-term classical stimulation with LPS rather than alternative activation. Therefore, TIPE2 can be a critical switch that negatively modulates arginine metabolism and rewrites classical M1 into an anti-inflammatory phenotype. On the other hand, iNOS is enhanced in M1 macrophages, resulting in the catabolism of arginine into citrulline and NO that are important for intracellular killing of pathogens. These results illustrate that examining the metabolic profiles of macrophages can develop more potential therapeutic target in switching phenotypes.

NO produced in murine macrophages is involved in the regulation of macrophage phenotypes via TCA cycle modifications and citrate accumulation. NO inhibits the TCA enzyme aconitase. Furthermore, the inflammatory macrophages reroute pyruvate far away from pyruvate dehydrogenase (PDH) depending on NO but not HIF-1*α*, consequently promoting glutamine anaplerosis and eventually leading to the suppression of ETC complexes [135].

#### 2.7.5. Fatty Acid Metabolism

An increased fatty acid synthesis is observed in LPS-stimulated macrophages [136]. Castoldi et al. found that triacylglycerol synthesis promotes the macrophage inflammation and decreases FA oxidation [137]. The inhibition of triacylglycerol synthesis significantly blocks lipid droplet (LD) formation, which further affects the production of PGE2, IL-6, IL-1*β*, and phagocytic capability. A recent report indicated that fatty acids are preferable substrate in prorepair M2 macrophages after cardiac injury [138]. An increased arachidonic acid metabolism and eicosanoid synthesis activated by the cyclooxygenase 2/1 (COX2/COX1) ratio, isoform of microsomal prostaglandin E synthase (mPGE2S), arachidonate 5-lipoxygenase, leukotriene A4 hydrolase, and thromboxane A synthase 1 are prominent in M1 macrophages. Conversely, COX1 and 15-lipoxygenase are stimulated, whereas the mPGE2S is inhibited in the M2 macrophage [139]. Moreover, TLRs also stimulate the production of nonclassical eicosanoids including resolvins and lipoxins, which support anti-inflammatory or proresolving activities [140]. An elevated FA metabolism and corresponding stimulation of PPAR-*α*/*β*/*δ* in macrophages seem to play a significant role in switching macrophages into a reparative phenotype. In humans, M1 macrophages induce glycolysis and result in the production of proinflammatory IL-6, IL-12, p40, and TNF-*α* similar to murines. However, the M2 phenotype oxidative metabolism and fatty acid oxidation are not prevailed instead of the gluconeogenesis induced by Fbp1 largely involved in macrophages [141]. The key enzyme fatty acid synthase (FAS) plays a critical regulatory role in M1 stimulation [142]. This study illustrates that the FAS is essential for the membrane remodeling, and the impaired FAS can cause alterations in the plasma membrane composition. The stimulation of FAS by the mitochondrial uncoupling protein 2 in macrophages also intervenes the activation of the NOD-like receptor (NLR) family pyrin domain containing 3 (NLRP3) inflammasome and the subsequent secretion of IL-1*β* and IL-18 in response to the LPS stimuli [143]. However, the mechanistic pathways of endogenous and exogenous FAs that induce inflammasome activation in macrophages are still unclear. A recent study shows that the saturated fatty acids rather than unsaturated fatty acids activate the inflammasome via increasing phosphatidylcholine levels and thereby cause suppression of membrane fluidity and subsequent disruption of Na^+^/K^+^ ATPase, which in turn leads to a K^+^ efflux [144].

Namgaladze and Brüne reported that in humans, IL-4-induced macrophages do not stimulate PGC-1*β*, an essential transcription factor required to induce fatty acid oxidation. In murine studies, blockers of fatty acid oxidation have no impact on the production of M2-type factors such as Mrc1 and CCL18 [145], indicating the principal variances in the metabolic needs of macrophage phenotype modifications between humans and mice. The fatty acid oxidation can indeed regulate the proinflammatory functions of macrophages. A recent study shows that larger intracellular levels of unsaturated fatty acids such as arachidonic acid, oleic acid, and linoleic acids rather than saturated fatty acids trigger the generation of IL-1*α* in foam cells, which in turn causes severe inflammation [146]. Particularly, the M2 macrophages activated with IL-4 depend on fatty acid oxidation through STAT6 and PGC1*β* and suppress inflammatory responses [117, 147]. The influence of FA oxidation on M2 macrophages is primarily noticed by reducing fatty acid oxidation via regulating AMP kinase, a probe responsible for metabolic alterations to elevate ATP levels, which in turn impairs the inflammatory resolution functions of macrophages [148].

#### 2.7.6. Amino Acid Metabolism

In recent years, the roles of amino acids in development and effector actions of immune cells have been considered especially for immune modulation [149]. L-Arginine is a crucial amino acid that can modulate immune cells. In M1 macrophages, L-arginine is metabolized into NO and citrulline by iNOS. In contrast, suppression of iNOS leads the M1 macrophage metabolic and phenotype switch towards M2 macrophages. By contrast, in M2 macrophages activated with IL-4, L-arginine metabolized by arginase-1 results in the production of urea and L-ornithine [90]. Thereafter, it serves as a precursor for the polyamines and proline that are required for the synthesis of collagen and tissue remodeling of the M2 macrophage [150]. Glutamine is another nonessential amino acid that significantly contributes to the polarization of M2 macrophages by activating the glutamine-UDPN-acetylglucosamine (GlcNAc) pathway to produce *α*-ketoglutarate [94]. On the other hand, succinate produced from the glutamine-dependent anerplerosis or *γ*-aminobutyric acid (GABA) triggers polarization of M1 macrophages [131]. Glutamine plays an important role in M2 macrophages by providing almost third portion carbon to replenish the TCA cycle. In contrast, suppression of glutamine synthetase swifts M2 macrophages towards M1 polarization, suggesting its integral role in the M2 macrophages [151]. However, the current data available on the glutamine metabolism in the regulation of macrophage phenotypes is largely from *in vitro* studies. Moreover, how they modulate the phenotype functions of the human macrophage is still unclear.

## 3. Unfavorable Inflammatory Microenvironment in Tissue Regeneration

Although the tissue repair and regeneration process critically involves the resolution of inflammation as well as tight control of immune response, this process is often dysregulated, leading to fibrosis and scar formation that disturb tissue architecture and functions.

### 3.1. Fibrosis

Fibrosis is a pathological state due to chronic and uncleared inflammation that triggers the production of synchronic inflammatory, regenerative, and angiogenic components in an uncontrolled manner [152]. Development of fibrosis ultimately leads to organ dysfunction and death. In response to the DAMPs and inflammatory factors of macrophages during injury, the inflammation and associated functions of fibroblasts will be enhanced to promote their migration, differentiation, and synthesis activities [153]. In general, they synthesize new proteins and ECM proteoglycan constituents to reconstruct a typical tissue structure. An excessive deposition of ECM during the process of fibrosis drastically impairs tissue structure and functions [46]. Wynn and Ramalingam hypothesized that constant stimulation and sustained mobilization of M(IL-4)-like cells may be involved in the production of pathological fibrosis [4]. Nonetheless, so far the majority of studies on fibrosis have engrossed on the inflammatory functions of macrophages. Murray et al. demonstrated that serum amyloid P (SAP) inhibits TGF-*β*1-induced pathologies including airway inflammation, apoptosis, pulmonary fibrocyte accumulation, and collagen deposition, without affecting TGF-*β*1 level [154]. In addition, SAP minimizes pulmonary M2 macrophages and enhances chemokine IP10/CXCL10 expression in a SMAD3-independent manner.

Fibrosis is predominantly accompanied by the crippled angiogenesis and persistent generation of local tissue hypoxia. The principal oxygen homeostasis regulator HIF-1*α* is instantly associated with the TGF-*β*1 during fibrogenesis [155]. Certainly, attenuation of HIF-1*α* expression significantly declines TGF-*β*1 generation in alveolar macrophages and impairs the production of bleomycin-induced fibrosis, substantiating the important role of TGF-*β*1 in the development of fibrosis. However, fibrosis can also be independent of TGF-*β* [156], wherein type 2 cytokine, namely IL-13, performs a major role in many cases [157]. The involvement of macrophages in the production and stimulation of IL-13 cytokine is still unclear because macrophages are thought to be not a major source of IL-13 [158]. An endoplasmic reticulum (ER) protein, disulfide isomerase containing thioredoxin domain 5 (TXNDC5), contributes to fibrogenesis by stimulating TGF-*β*1 signaling cascade through binding and stabilizing lung TGFBR1. Furthermore, activation of TGF-*β*1 upregulates TXNDC5 through an ER stress/ATF6-dependent regulation of lung fibroblasts [159]. A transcription factor JUN plays a crucial role in the lung fibrogenesis by increasing the expression of CD47, programmed death-ligand 1 (PD-L1), and IL-6. The inhibition of CD47, PD-L1, and IL-6 reverses fibrosis by enhancing phagocytosis of fibroblasts and removing suppressive functions on the adaptive immune system [160]. The oxidative stress-induced hepatocyte premature senescence implicated in liver fibrosis is attenuated by IGF-1 via promoting cytoplasmic Akt1-p53 interaction, which in turn blocks nuclear p53-progerin (farnesylated mutant lamin A protein) interaction [161]. A bioactive chitosan hydrogel prepared with immobilizing the C domain of IGF-1 peptide (IGF-1C) and adipose-derived mesenchymal stem cell (ADSCs) cotransplantation against ischemic kidneys attenuates fibrosis and ameliorates renal functions [162]. Therefore, IGF-1 embracing paracrine effects may be a promising therapeutic target to reduce fibrosis. Perhaps, the activated macrophages trigger fibrosis by producing cytokines that stimulate fibroblasts to produce collagen and ECM. The activated profibrotic myeloid cells solely express folate receptor *β*. The folate-targeted TLR7 agonist (FA-TLR7-54) rewrites M2 similar cells that produce fibrosis cytokines. This results in the suppression of profibrotic cytokine secretion, biosynthesis of hydroxyproline, deposition of collagen, and consequent expansion of alveolar airspaces [163]. One challenge for the regenerative investigators is to regulate phenotypes of fibroblasts and mesenchymal cells that orchestrate tissue regeneration. In this regard, novel strategies using biomaterials enriched with growth factors, stem cells, and decellularized ECM scaffolds can significantly improve the quality of regenerated tissues.

### 3.2. Chronic Inflammation

Chronic inflammation is characterized by the prolonged response to inflammatory signals involved in continuous recruitment of lymphocytes, monocytes, and macrophages with neovascularization as well as connective tissue proliferation. Chronic inflammation sustains tissue remodeling and impairs tissue functions in several diseases. It is evident that the resolution phase of inflammation encompassing the control of extravasation of immune cells, regulation of production of chemokines and cytokines, shutdown of signaling pathways connected to leukocytes, and succeeding leukocyte removal via efferocytosis after an injury is essential to restore tissue homeostasis. However, an impaired resolution of inflammation causes chronic inflammatory diseases such as colitis and asthma and is also involved in immutable tissue damage as well as enhanced risk for the development of cancer, osteoporosis, and cardiovascular diseases [164–166]. Unlike the acute inflammation that is initiated by the DAMPs and PAMPs, the systematic chronic inflammation is classically triggered by DAMPs in the absence of PAMPs [167, 168]. Therefore, the development of drugs and therapies is chronically weighed on anti-inflammatory steroids, nonsteroidal anti-inflammatory drugs (NSAIDS), and furthermore targeted strategies including TNF monoclonal antibodies.

In this review, we insinuate that novel approaches that mitigate the negative functions of inflammation in a tissue microenvironment may allow constructive functions in the immune response. Another systematic emerging idea is to regulate the polarization of immune response, and the contemporary data also indicate that modulating the entry port of inflammatory cells could be one scheme, although macrophages may also activate matrix metalloproteinases (MMPs) and other degenerative enzymes to affect ECM. Several types of MMPs are involved in the clearance of fibrosis, but some others function in the development of fibrosis. The chronic inflammation-associated molecular composition of diseases and current therapies are reviewed in detail in the following section.

## 4. Chronic Inflammation-Associated Diseases and Therapies

### 4.1. Myocardial Infarction (MI)

MI is a type of acute coronary syndromes caused by acute and persistent ischemia and hypoxia [169] and has recently become one of the leading causes of death and disability. In recent years, the clinical therapeutic methods of MI mainly contain pharmaceutical therapies for thrombolysis, antiplatelet and antihypertension, and interventional therapies such as percutaneous coronary intervention (PCI) and coronary artery bypass grafting (CABG) [170]. Longer time of coronary artery blockage leads to a larger area of irreversible myocardial necrosis and finally death. Therefore, the patients with acute MI should be treated with thrombolysis as soon as possible to make the infarcted blood vessels reopen completely and continuously for saving the dying myocardium, preserving the cardiac function, and reducing mortality [171]. Thrombolytic drugs include streptokinase, urokinase, and recombinant streptokinase. Urokinase is widely used due to the absence of antigenicity, definite curative effect of turning plasminogen into plasmin to degrade fibrin and dissolve thrombus, and convenient application without a skin test [172]. Compared to the conservative and single pharmaceutical therapies, interventional therapies are universally considered more effective MI reperfusion methods for further reducing mortality from 10% to 5% [173]. However, the slow flow/no-reflow phenomenon occasionally occurring after the interventional therapies leads to ineffective treatments, which remains a challenge and difficulty due to its undefined mechanism [174]. Advances have been achieved recently in the possible mechanisms that are related to the fracture and shedding of the thrombus tissue during the mechanical compression of the balloon or stent, resulting in microvascular damage, microcirculation embolism, microvascular spasm, reperfusion injury, inflammation, release of ROS, and/or swelling of cardiomyocytes [175]. To deal with this phenomenon, preoperative and intraoperative application of some drugs such as tirofiban, alprostadil, and nitroprusside together with a thrombus aspiration device makes much sense for inhibiting microcirculation embolism and promoting microvascular expansion to some degree [176–179].

Considering that the clinical therapeutic methods still have some limitations in irreversible myocardial necrosis and declined cardiac function, other effective regeneration medicine strategies including stem cell therapy [180, 181] and regenerative biomaterial (such as nanoparticles, patches, scaffolds, and hydrogels) therapy have been developed recently. In particular, the ROS-responsive biomaterials play an important role in the tissue microenvironment and regeneration [182]. For example, implantation of thioketal- (PUTK-) based ROS-responsive polyurethane patches loaded with glucocorticoid methylprednisolone (MP) improved significantly the reconstruction of functions of the myocardium including elevated ejection fraction, reduced infarction size, and increased revascularization in MI rats [183].

Inflammation participates in and plays a key role in the pathophysiological process of heart damage, repair, and regeneration after MI [184–186], which provides a basis and guidance for regenerative medicine strategies. Generally, initial ischemia and hypoxia after MI result in massive cell apoptosis, and the released DAMPs jointly induce acute inflammation in the tissue microenvironment [187]. Then, the immune cells such as neutrophils and macrophages are recruited successively to secrete more inflammatory factors such as TNF-*α*, IL-6, and IL-1*β*, which further exacerbate the inflammation and abnormal ventricular remodeling in the MI area [21]. Stem cells are a type of cells with high plasticity and self-renewing ability and are applied in a large number of clinical studies. Efficiency of stem cell therapies in MI is mainly based on two hypotheses: differentiation into myocardial tissue-related cells such as cardiomyocytes and paracrine effect. Lee et al. injected 2 million ADSCs into the coronary artery in a pig MI model [188]. The ADSC treatment group could increase the capillary density and reduce the infarction area to improve the final cardiac function, likely due to the differentiation of ADSCs into vascular endothelial cells instead of cardiomyocytes. Gnecchi et al. found that implanted bone marrow-derived mesenchymal stem cells (BMSCs) are able to activate endogenous stem cells and promote the proliferation of original cardiomyocytes through a paracrine effect to achieve the inhibition of MI [189]. However, simple injection of stem cells faces some inevitable problems such as low cell retention, time-consuming cell preparation, and potential allogeneic immune response [190]. Hao et al. fabricated an injectable fullerenol/alginate hydrogel loaded with ADSCs for cardiac repair [180]. They found that fullerenol nanoparticles can scavenge excessive ROS to reduce microenvironment inflammation and then improve the survival capacity of ADSCs, which are beneficial for increased vascularization and cardiac function. Moreover, the direct usage of noncell regeneration medicinal biomaterials having a property of modulating the MI inflammatory environment presents a greater potential and convenience for MI treatment. Han et al. synthesized graphene oxide (GO) particles of 150 nm loaded with IL-4 plasmid DNA (IL-4 pDNA) to treat MI [191]. The GO particles can reduce inflammation as an antioxidant, and the IL-4 pDNA increases the ratio of anti-inflammatory M2 macrophages to inflammatory M1 ones, leading to significant myocardial repair. Fan et al. prepared a glutathione- (GSH-) modified collagen hydrogel loaded with recombinant protein glutathione-S-transferase- (GST-) TIMP-basic fibroblast growth factor (bFGF) [192]. TIMP is a peptide PLGLAG that can respond to upregulated MMP-2/9 and break to release GST and bFGF on demand, which inhibits the degradation of ECM by MMP-2/9 and contributes to the final vascularization and MI repair.

In summary, the most clinically used treatment for MI is PCI therapy together with thrombolytic drugs and a thrombus aspiration device for better reperfusion efficiency. Considering the limitations of irreversible myocardial necrosis and declined cardiac function of clinical methods, regenerative biomaterial therapies deserve more attention.

### 4.2. Atherosclerosis

**Figure 4:**
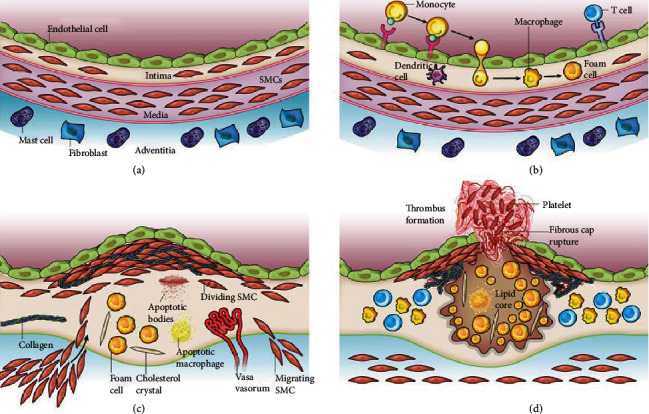
(a, b) Stages in the development of atherosclerotic lesions. Reprinted with permission from [195] Copyright © 2021 Springer Nature.

Atherosclerosis is a chronic inflammatory vascular disease, characterized by lipid deposition and fibrosis underneath the inner wall of vessels, which may result in serious clinical symptoms such as sudden cardiac death, acute myocardial infraction, and stroke [193]. Atherosclerosis-related inflammation is associated with immune activation and induction of inflammatory mediators and signaling pathways [194]. The formation of atherosclerosis is a continuously changing process, and the pathological development progress mainly includes three stages. The inflammation plays an important role in all stages of the atherosclerotic process (Figure 4(a)) [195]. In the first stage, the accumulation of low-density lipoprotein (LDL) on the arterial wall takes place and further passively diffuses via endothelial cell (EC) junctions, leading to pathological intimal thickening [196]. In the nascent atheroma, monocytes can be observed adhering to the surface of the endothelium. With time prolongation, the monocytes pass through the endothelial monolayer to the intima, where they proliferate and differentiate into macrophages and foam cells (Figure 4(b)). Macrophages in the atheroma may also have the characteristics and probably the antigen-presenting functions. During atherogenesis, the smooth muscle cells (SMCs) migrate from the media into the intima and produce ECM molecules such as collagen and elastin (Figure 4(c)). Then, the fibrous cap ruptures to trigger thrombus. Therefore, the therapies of atherosclerosis in its early stage play an important role in slowing down the lesions and saving the lives of patients.

The promising therapeutic strategies include nonspecific anti-inflammatory drugs such as allopurinol, colchicine, methotrexate, and aspirin; biologic therapies targeting chemokines and cytokines such as tumor necrosis factor inhibitors and IL-1 neutralization; and small-molecule enzyme inhibitors such as phospholipase inhibitors and antileukotrienes, as well as targeting of inflammatory signaling pathways (inhibition of NADPH oxidase, p38 mitogen-activated protein kinase (MAPK), or phosphodiesterase) [197]. Simultaneously, medications to lower lipid, anticoagulation, hypertension control, and prevention of thrombosis as well as antiplatelet drugs are used as routine treatments for antiatherosclerosis [198].

However, severe atherosclerosis always induces a large area of lumen occlusion, which requires surgical intervention such as the technique of percutaneous coronary intervention (PCI) or coronary artery bypass grafting (CABG) surgery [199]. Considering that stents are unsuitable for those with small or tortuous vessels or lesions at vessel bifurcation and high risk of restenosis, their widespread use may be limited. CABG surgery is the standard of care for patients, having the better long-term patency and showing a reduction in morbidity and mortality compared to PCI. However, the mortality, duration of operation, and risk of sternal wound problems are reported for this type of graft [200]. Both carotid endarterectomy (CEA) and carotid artery stenting (CAS) are the standard treatment for carotid atherosclerosis [201].

**Figure 5:**
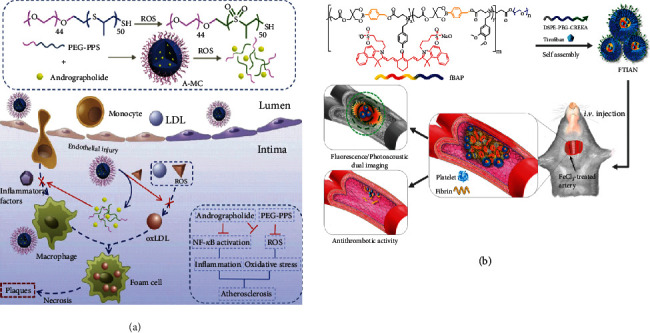
(a) Schematic illustration of the therapeutic mechanism by PEG-b-PPS micelles for reducing ROS and proinflammatory cytokines in the atherosclerotic process. Reprinted with permission from [203] Copyright 2018 Elsevier Ltd. (b) Schematic diagram of FTIAN as a thrombus-specific theranostic agent that is able to image thrombus and exert potent antithrombotic activity. Reprinted with permission from [204] Copyright © 2021 American Chemical Society.

Nanocarriers or polymer-based therapeutics used in the context of atherosclerosis treatment have been widely reported, which can overcome the limited efficacy and plenty of side effects related to the existing strategies based on conventional drug delivery systems [202]. Inflammatory signaling plays an important role in atherosclerosis and is one of the most promising targets for the nanosystems. For example, the ROS-responsive PEG-b-PPS micelles are used as a smart drug delivery system to treat atherosclerosis, which not only quickly release the encapsulated drug and rographolide but also consume ROS by themselves. The micelles can simultaneously decrease the inflammatory response and ROS level to treat atherosclerosis (Figure 5(a)) [203]. Moreover, by taking the physicochemical and biological characteristics of thrombus, fibrin-targeted and H_2_O_2_-responsive nanoparticles have been developed (Figure 5(b)) [204]. A fibrin-targeted imaging and antithrombotic nanomedicine (FTIAN) can target fibrin specifically to image thrombus, scavenge H_2_O_2_, and prevent platelet activation, which can reduce the formation of thrombus. Consequently, the inflammatory response biomaterials combined with different drugs can be a more effective antiatherosclerotic therapy and deserved further study.

### 4.3. Inflammatory Bowel Disease

**Figure 6:**
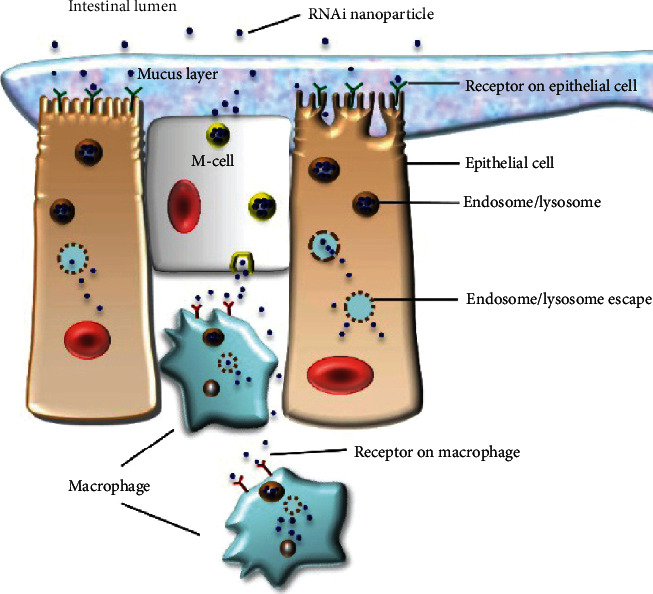
siRNA nanoparticles target epithelial cells or macrophages in the intestinal lumen. Reprinted with permission from [210] Copyright © Dove Press 2016.

Inflammatory bowel disease (IBD) is a chronic inflammatory disorder of the gastrointestinal tract, which can be divided into chronic relapsing inflammatory disorders (Crohn's disease) and ulcerative colitis. IBD is considered an inappropriate and continuing inflammatory response to commensal microbes in a genetically susceptible host [205]. Nonsteroidal anti-inflammatory drugs and corticosteroids as well as immuno-suppressive and immuno-regulatory agents such as methotrexate, azathioprine, and its metabolite 6-mercaptopurine are used to cure IBD, aimed at reducing intestinal inflammation and immune system hyperresponsiveness [206]. Besides, a selective TNF-*α* blocker, the monoclonal antibody infliximab, is considered a major advance in IBD therapy [207]. Conventional oral formulations are limited for use in IBD due to the adverse effects and toxicity following distribution of drug among the body [208]. Compared with the conventional agents, small interfering RNA (siRNA) can effectively alleviate IBD progression and promote intestinal mucosa recovery through precise regulation of the expression of proinflammatory cytokines related to IBD [209]. Nanoparticle delivery systems for siRNA confer stability of RNA during delivery *in vivo*, and the nanoparticles with ligand modification can target macrophages in the inflamed intestinal mucosa [210] (Figure 6).

For the treatment of IBD, nanoparticles show special advantages including protecting drugs and increasing drug release/retention at diseased sites. The nanoparticle delivery systems include liposomes and polymer-based nanoparticles [210]. For instance, a kind of nanoparticles formulated from ROS-responsive poly(1,4-phenleneacetonedimethylene thioketal) (PPADT) is synthesized [211]. The NPs successfully release loaded TNF-*α*-siRNA that knocks down the proinflammatory cytokine TNF-*α* in response to the high ROS level in the inflammatory gut region. A nanosystem designed based on Tempol (Tpl) and a biocompatible *β*-cyclodextrin-derived material (Tpl/OxbCD NP) was reported [212]. This nanomedicine is stable in the gastrointestinal tract and can effectively scavenge multiple components of ROS and selectively release Tpl in the inflamed intestinal tissues. Another pH and ROS-sequential responsive nano-in-micro composite for targeted therapy of IBD can selectively release rifaximin (an intestine-specific antibiotic, RIF) to the inflamed tissues [213].

### 4.4. Chronic Diabetic Wound

Millions of people get diabetes worldwide, and about 25% of them suffer from diabetic foot ulcer (DFU), which is a typical chronic wound and can lead to amputation [214]. Common acute wound caused by mechanical factors can heal naturally via a series of continuous and overlapping physical stages such as haemostasis, inflammation, proliferation, and remodeling [215]. But the chronic wound can hardly recover because of its abnormal wound microenvironment. The chronic wounds are confirmed by persistent inflammation, impaired angiogenesis and epithelialization, disordered cytokine/growth factor secretion, and excessive degradation of ECM, which delay the normal progression of wound healing [216]. The common features of the chronic diabetic wounds are high level of ROS [217], formation of bacterial biofilms [218], excessive neutrophil infiltration [219], and the maladjustment of the number of different macrophage phenotypes in the wound [62]. Aimed at these symptoms in diabetic wounds, various strategies have been developed to adjust the chronic inflammation and accelerate skin regeneration.

The nanoparticles have been extensively used for wound healing, which may have different functions such as anti-infection, antioxidant, immunoregulation, and controlled release of drugs/cytokines/genes [220]. Qiao et al. prepared copper sulfide nanodots that can eradicate multidrug-resistant bacteria owing to the photothermal effect [221]. Meanwhile, the controlled release of copper ions can accelerate wound healing by promoting fibroblast migration and endothelial cell angiogenesis. The excessive ROS in chronic wounds lead to severe damage to nucleic acids and proteins [222]. Liu et al. developed a simple and efficient one-step way to get ultrasmall Cu_5.4_O nanoparticles. This nanoparticle has multiple enzyme-mimicking properties and can scavenge broad-spectrum ROS, which is beneficial to wound healing [223]. Gan et al. prepared a kind of konjac glucomannan-modified SiO_2_ nanoparticles with immunoregulation function, which can reduce the inflammation level and promote wound healing by inducing macrophages to differentiate into M2 phenotypes *in situ* [224]. This material provides a new endogenous regulation method to treat diabetic wound without adding drugs or exogenous M2 phenotype macrophages. Due to their unique biochemical characteristics, lipid-based vesicles are applied to the constructions of an advance drug delivery system. A recent study indicates that dexamethasone-loaded liposomes can achieve local delivery to primary human macrophages, induce an anti-inflammatory/proresolution phenotype, and promote diabetic wound healing [225].

Hydrogels are three dimensional networks of hydrophilic polymers filled with water [226]. Due to their high hydration (similar to native ECM), tunable properties, and porous architecture, they are highly recognized in biomedical applications [227]. A gelatin methacryloyl- (GelMA-) based adhesive hydrogel for the local delivery of miR-223 5p mimic (miR − 223∗) encapsulated in hyaluronic acid- (HA-) based nanoparticles was developed [228]. This hydrogel delivery system with miR − 223∗ efficiently promotes the formation of uniform vascularized skin at the wound site, which is mainly due to the polarization of macrophages to the M2 phenotype. Zhao et al. prepared a multifunctional hydrogel (PDA@AgNPs/CPHs) with many desirable features, including antibacterial property, tunable mechanical and electrochemical properties, repeatable adhesiveness, good processability, and self-healing ability [229]. This hydrogel shows a significant effect on promoting angiogenesis, accelerating collagen deposition, inhibiting bacterial growth, and controlling wound infection in a diabetic wound healing animal test. Besides, this conductive hydrogel is a biocompatible detector that can monitor movements of the human body in real time. Xiao et al. designed an antioxidant thermoresponsive citrate-based hydrogel (H-HKUST-1) embedded with copper metal organic framework nanoparticles, which can decrease toxicity of copper ion and accelerate diabetic wound healing [230]. The animal test result shows that the H-HKUST-1 hydrogels can induce angiogenesis, collagen deposition, and reepithelialization during wound healing.

Electrospinning technology provides a convenient and direct approach to prepare scaffolds consisting of microfibers/nanofibers [231]. Various types of electrospun fibers such as core-shell structure [232] and hollow [233] structure have been prepared so far. Since the microfibers/nanofibers possess a high specific surface area and a highly porous 3D network, the electrospun nanofiber scaffolds have a well interaction with cells [234]. Different chemical structures and modifications also endow the fibers with plenty of functions [235]. Augustine et al. reported the development of electrospun poly(3-hydroxybutyrate-co-3-hydroxyvalerate) (PHBV) membrane incorporated with cerium oxide nanoparticles (nCeO_2_) for diabetic wound healing application [236]. The nCeO_2_-PHBV membranes can enhance cell proliferation and vascularization to promote the healing of diabetic wounds. Liu et al. prepared a kind of electrospun thioether-grafted hyaluronic acid nanofibers (FHHA-S/Fe) that can form a nanofibrous hydrogel *in situ* on the wound, which combine the advantages of both hydrogel and nanofibers [237]. The *in vivo* test shows that this material can synergistically modulate the inflammation microenvironment to help diabetic wound healing by scavenging ROS effectively in the early inflammation phase and promoting transformation of the gathered M1 macrophages to the M2 phenotype.

Dysfunction of HIF-1*α* is one of the reasons that hinder the healing of diabetic wound [238]. Gao et al. prepared poly(*ε*-caprolactone)/type I collagen electrospun nanofiber wound dressings loaded with dimethyloxalylglycine (DMOG), which can stabilize HIF-1*α* by inhibiting its degradation [239]. The *in vivo* test shows that the DMOG-loaded nanofibers can improve diabetic wound healing by accelerating reepithelialization, angiogenesis, and wound closure.

The electrospun fibers can also be applied to deliver peptides. Liraglutide (Lira), a GLP-1R receptor agonist, has been reported to promote the angiogenic ability of endothelial cells [240]. Yu et al. prepared a poly(lactide-co-glycolide)/gelatin (PLGA/Gel) nanofibrous membrane that achieves controlled release of liraglutide [241]. The PLGA/Gel/Lira can shorten wound closure time and increase blood vessel density, collagen deposition, and alignment, efficiently improving the healing process of diabetic wounds.

In summary, the recent studies show that more and more biomaterials for diabetic wound healing attach importance to wound microenvironment adjustment, including the regulation of inflammation, cytokines/nucleic acids, phenotypes of macrophages, and elimination of excessive ROS/reactive nitrogen species (RNS). Instead of delivery of drugs or growth factors directly, a suitable wound microenvironment can activate the natural wound healing process correctly, which involves a series of exquisitely complex physiological activity.

### 4.5. Osteoarthritis

Osteoarthritis (OA) is a whole joint disorder that involves structural changes in hyaline articular cartilage, subchondral bone, ligaments, capsules, synovium, and muscles around the joints [242], which is the most prevalent joint disease afflicting millions of wordwide people [243, 244]. Accumulating research results indicate that chronic low-grade inflammation is the key to the pathological process and symptoms of OA [245], instead of the traditional view that osteoarthritis is only a degenerative disease. The pathogenesis of osteoarthritis is quite complicated, including many factors such as mechanics, inflammation, and metabolism [242].

In OA joint tissue and synovial fluid, not only are the levels of plasma proteins, complement components, and cytokines abnormally high but also the chondrocytes and synovial cells are activated to overproduce redundant inflammatory mediators [246] such as IL-1 and TNF-*α*, prostaglandins, NO, and much too high concentration of ROS [247]. The overexpressed inflammatory mediators in response to joint structural breakdown alter metabolism and differentiation behaviors of chondrocytes, inducing secretion and activation of MMPs [248] and aggrecanases [249], both of which can degrade the ECM. The abnormality of a variety of cells and inflammatory mediators worsens the inflammatory microenvironment of osteoarthritis, disrupts the dynamic balance in the joints, and might eventually lead to joint failure.

Nonsurgical therapies such as physical treatment, anti-inflammatory drugs, analgesic or chondroitin sulfate supplementation, and local injection of sodium hyaluronate are firstly adopted to temporarily alleviate pain and relieve symptoms for the early OA [242], which are difficult to inhibit the progress of the disease and completely repair the damaged cartilage. Early diagnosis and timely treatment of OA play critically important roles in delaying the onset and progression of severe OA, of which the treatments have to rely on surgical interventions [243, 244] such as joint replacement, marrow stimulating techniques, and joint debridement. Since all of these surgical procedures have defects, tissue engineering has led to the development of more advanced regenerative techniques [250]. Emerging treatments combined with biologicals, ranging from growth factors, blood derivatives, and genes to stem cells or chondrocyte transplantation and stem cell-derived exosomes, are advocated as the promising tools, which make a difference in regulating immune function, lowering inflammatory mediators, and thus reducing joint damage and promoting cartilage regeneration [251, 252].

Various types of biomaterials including hydrogels [253–255], nanoparticles [256–260], electrospun membranes [261], 3D printed materials [262], drug or bioactive factor carriers, and tissue engineering scaffolds have been applied for the treatment of OA. In particular, the hydrogel has been deemed as an attractive option, attributed to the capabilities to stimulate the ECM environment and to incorporate bioactive molecules and drugs as well as the convenience of handling and minimally invasive repair of cartilaginous tissues via adjusting chemical structure, injectability, and crosslinking properties [263]. Madry et al. have prepared an injectable hydrogel based on thermosensitive poloxamers, which is loaded with and able to release a therapeutic recombinant adeno-associated virus (rAAV) vector overexpressing the chondrogenic SRY-box transcription actor 9 (SOX9) in full-thickness chondral defects [253]. The hydrogel-virus complex is applied in a clinically relevant minipig model *in vivo*, which may represent a major step towards improved cartilage repair in the near future. Besides, the hydrogel scaffolds with the capabilities to promote cartilage tissue repair based on regulating the cartilage microenvironment present a novel perspective for cartilaginous tissue regeneration. Feng et al. have fabricated an aggrecanase-1 cleavable hydrogel conjugated with N-cadherin mimetic peptides HAVDIGGGC to mediate the degradation of hydrogels and cell-cell interactions of encapsulated BMSCs, thus achieving a better regeneration of osteochondral defects [254].

The intra-articular treatment systems in virtue of injectable hydrogels and nanobiomaterials provide effective long-term symptom relief and disease-modifying properties via enhancing whole joint retention and regulating the OA inflammatory microenvironment, especially for the management of the early or midstage OA [264]. For instance, Jin et al. have developed a hyaluronate acid-based injectable hydrogel physically mixing epigallocatechin-3-gallate to scavenge ROS, which inhibits inflammation and enhances cartilage regeneration [255].

Recently, nanoparticles have been modified through active and passive targeting strategies to facilitate localization and interactions with specific joint tissues such as cartilage and synovium [265]. In this way, the therapeutic system based on nanoparticles with the capabilities of regulating the OA microenvironment can intelligently enter the targeted cells and thereby efficiently suppress inflammation and delay the progression of OA. Li et al. have demonstrated that polyethyleneimine-modified mesoporous silica nanospheres can successfully bring hyaluronan synthase type 2 into synoviocytes to synthesize endogenous HA with high molecular weight and downregulate the synovial inflammatory mediators in the OA microenvironment, hence promoting the self-repairing mechanism and putting forward a totally different method for OA management [258]. Chen et al. designed a novel MMP-13/pH-responsive and cartilage-targeting ferritin nanocages encapsulating hydroxychloroquine for OA imaging and therapy, which can smartly monitor the overproduced MMP-13 in OA and release hydroxychloroquine induced by weak acid. Such theranostic nanoplatform holds promise for smart and precise OA clinical application [259]. In addition, specifically targeting and regulating macrophages are other crucial sources of inflammatory responses in OA joint, representing a fresh trend towards therapeutic platform construction. Liang et al. have fabricated a multifunctional anti-inflammatory drug delivery system based on a carbonic oxide (CO) release molecule loaded in peptide dendrimer nanocages, the surfaces of which are wrapped with folic acid- (FA-) modified HA [261]. The system can be smartly delivered into activated macrophages via FA- and HA-mediated specific targeting effects and then rapidly release CO by depleting hydrogen peroxide to inhibit the expression of IL-1*β*, IL-6, and TNF-*α*, thus to inhibit the degradation of articular cartilage ECM.

In summary, OA is still an incurable joint disease. The ever-improving biomaterial-based OA therapeutic platforms have been designed to effectively regulate the OA inflammatory microenvironment to suppress the secretion of inflammatory mediators, relieve symptoms, and promote joint tissue repair and regeneration, which turns out to be highly efficient options for OA management. Besides, the emerging therapies via targeting and regulating the key inflammatory cells to improve the OA microenvironment provide new insight into OA management and hold promise on the precise treatment of every stage of OA.

### 4.6. Acute Lung Injury (ALI)

Inflammation is the body's defence against internal and external harmful stimuli. However, uncontrolled inflammation can injure cells, tissues, and organs and cause damage to the body [266]. Acute lung injury (ALI) is diffuse alveolar damage induced by excessive inflammatory response, which may further develop into acute respiratory syndrome (ARDS) and thereby threaten to life health if not timely treated [267, 268].

Various internal and external injury factors such as tissue necrosis, bacteria, and viruses can cause damage to epithelial cells of alveoli. The macrophages in alveoli will recognize these factors and release inflammatory mediators such as TNF-*α* and IL-1 that can activate capillary endothelial cells and recruit white blood cells (WBCs) such as neutrophils and monocytes from the blood vessel and bone marrow to the inflamed tissue [269]. The activated endothelial cells will overexpress intercellular adhesion molecular-1 (ICAM-1) that can bind to integrin expressed on WBCs' membrane and then help WBCs migrate and adhere onto the vascular wall. Meanwhile, endothelial cells will shrink under the stimulation of inflammatory mediators, leading to the increase in vascular permeability [270], followed by the WBCs and protein edema fluid exuding to the alveoli. More WBCs will gather in the inflammatory site because of chemotaxis and are activated by the inflammatory mediators. Furthermore, the activated WBCs will take the phagocytosis role and release ROS and lysosomal enzymes to kill and degrade the pathogens. If the pathogens cannot be eliminated in a short time, the WBCs will not stop to migrate to the infected tissue and release more ROS and lysosomal enzymes not only within the cells but also outside cells, resulting damage to normal lung tissue and cells. Therefore, there are high levels of cytokines and chemokines such as ROS, lysosomal enzymes, TNF-*α*, IL-1, and more WBCs especially neutrophils in the ALI microenvironment.

There are no effective methods to treat ALI at present, because the available drugs with a high dose are poorly targeted to the lung and thus bring side effects on other organs [271]. When ALI is caused by bacteria or viruses, antibiotics and antiviral drugs are widely used in clinic. However, the multidrug-resistant bacteria and new viruses (such as SARS-CoV-2) are huge challenges [272, 273]. Apart from symptomatic treatment, supportive care is also important for recovery, including lung protection ventilation, nutritional support, and fluid management [274]. Therefore, anti-inflammatory therapy for pneumonia and lung injury is a necessary and effective method.

Nowadays, the drug delivery systems (DDS) that respond to the ALI microenvironment have attracted much concern [275]. Wang et al. used 4-(hydroxymethyl) phenylboronic acid pinacol ester-modified alpha-cyclodextrin (Oxi-alpha CD) as a carrier to obtain a kind of ROS-responsive moxifloxacin- (MXF-) loaded nanoparticles, which release MXF triggered by H_2_O_2_ and are effective in a mouse model of pulmonary *P. aeruginosa* infection [276]. Zhang and his coworkers developed an acid-sensitive nanoparticle to target inflamed lungs. The nanoparticle is composed of a low pH-sensitive polymer, poly(beta-amino esters), as a core for drug loading and controlled release, and its surface is modified with anti-ICAM-1 antibodies for targeting the lung. This kind of nanoparticles can target the inflamed site and release the loaded drug, reducing inflammation in an ALI mouse model [277]. Ma et al. provided a DDS that can not only responsively release drug but also have diagnosis property. The anti-inflammation drug prednisolone is conjugated to a two-photon AIE bioimaging compound via ROS-responsive linker, which is further encapsulated into a core-shell micelle. The micelle is composed of an amphipathic polymer that can release the drug through hydrophobic-to-hydrophilic transition of the core triggered by ROS [278]. In addition, our lab also prepared PPADT nanoparticles loaded with FK-506, which can suppress the lung inflammation caused by PM 2.5 [279].

The polymer-based DDS has good targeting property, which can reduce dose and side effects to drugs. Furthermore, the stimulus responsiveness enables the drugs to be released in a controlled manner and also eliminates inflammatory mediators in the lesion site, offering a promising therapy for the treatment of ALI.

### 4.7. Inflammatory Disorders of the Central Nervous System

The inflammatory-related diseases of CNS include a wide spectrum of inflammatory or autoimmune disorders of CNS distinguished by the immune-mediated tissue injury and existence of inflammatory infiltrations in the brain and spinal cord [280, 281]. The brain injury milieu is allied with immune reactions, i.e., the activation of innate immunity comprised of resident monocytes, astrocytes, and microglia as well as released cytokines and chemokines, and recruited macrophages, granulocytes, and lymphocytes from circulation. Although convincing evidence suggests that the adaptive immune cells are activated by the native antigen recognition, the consequent multiplication and clonal development are highly absent. The acute and chronic inflammatory brain disorders are predominated by the class I MHC-restricted CD8^+^ T-lymphocytes [282]. At later stages, astrocytes turn up in a reactive state to create glial scar tissue [283]. In general, CNS has minimal capacity to naturally regenerate after traumatic injury/disorders. Therefore, advanced tissue regenerative therapies are required to promote tissue and functional repair of CNS. One such strategy is transplantation of cells including mesenchymal stem cells (MSCs), neural stem cells, induced pluripotent stem (iPS) cells, and endothelial progenitor cells [284–287]. Although cell transplantation is largely preferred to regenerate CNS, several limitations remained due to less cell viability, lower cell survival, and uncontrolled cell multiplication and integration [288].

The conventional delivery of therapeutic molecules by oral and intravenous administration has also been restrained by diffusion over the blood-brain/spinal cord barrier [289]. By contrast, the composite strategies integrating cells, bioactive molecules, and biomaterials have thus attracted significant interest over the past few years to improve cell survival, reduce side effects across the blood-brain barrier, and achieve local delivery. For example, implantation of porous collagen-based scaffolds (PCSs) seeded with neural stem cells can enhance cell delivery and differentiation, activate robust axonal elongation, and decrease astrogliosis in animals [290]. HA is an extensively considered and modified natural polymer for a regenerative scaffold of CNS because it plays an important role in neural tissues by regulating cell migration, proliferation, and differentiation. For instance, HA interacts with the CD44 cell surface receptor of glial and neuronal cells, which regulates cell behavior at tissue damage conditions [291]. Moreover, HA-CD44 pairing in astrocytes triggers the Rac 1-dependent PKN (protein kinase N) pathway, leading to enhanced astrocyte migration, and activated Src family kinases (SFKs) and cascade of focal adhesion kinase (FAK) [292, 293]. Combination of HA with synthetic peptides in particular RGD and IKVAV potentially supports differentiation of neural progenitor cells (NPC) into oligodendrocytes and synapse-forming neurons [294]. Hydrogels have been employed to recapitulate and improve regeneration and functions of CNS [295]. Injectable self-assembling peptide-based hydrogel (SAPH) used directly on the rat brain after traumatic brain injury significantly triggers VEGF-receptor 2 and thereby enhances angiogenic effect and new blood vessel formation. Furthermore, the von-Willebrand factor (vWF) and *α*-smooth muscle actin (*α*-SMA) also increase along with blood vessel density [296]. Coupled self-assembling peptide (SAP) and neural stem/progenitor cell (NSPC) transplantation enhances engraftment of NSPC, synaptic connectivity, and behavioral outcomes and reduces CSPG deposition and astrogliosis [297]. The composite 3D biomimetic CNS scaffolds consisting of polyethylene glycol-gelatin-methacrylate (PEG-GelMa) can promote the repair of rat spinal cord injury [298]. The NSPS derived from the spinal cord are suspended in a fibrin matrix consisting of brain-derived neurotrophic factor, VEGF, fibroblast growth factor, and calpain inhibitor, which are loaded into a scaffold and then incorporated into a thoracic cord transection lesion of rats. After implantation, the 3D scaffold maintains its architecture for 6 months, the engrafted NCPS survive, the axons expand into the host spinal cord, and the synaptic transmission recovers, leading to electrophysiological and functional development. These studies show that with the suitable combination of ideally engineered scaffolds, stem cells, and growth factors, the adverse environment of brain/spine cord injury can be improved, resulting in the regeneration of CNS. Moreover, the use of electrically conductive biomaterials and integration of different functional scaffolds/hydrogels are more appreciable to achieve better neural tissue repair and regeneration.

## 5. Conclusions and Future Perspectives

The primary role of the inflammatory microenvironment particularly immune cells at the tissue injury/damage site is to establish and orchestrate proregenerative milieu. Different types of immune cells and several immune regulators participate in all the phases of tissue repair/regeneration and homeostasis. As discussed in this review, among diverse types of immune cells, monocytes and macrophages are critical players, which secure many functional traits that are critical for the tissue regeneration. However, the mechanistic stimulations by which they modulate phenotypes for proinflammation, anti-inflammation, prorepair, and profibrogenic mechanisms remain unclear. Further efforts are needed to fully understand the functional characteristics of distinct macrophage phenotypes in several organs.

The immune responses in potential tissue regeneration are crucial to regulate the disease environment. One such emerging strategy is the modulation of macrophage phenotype responses. Development of therapies specially targeting the immune system is restrained because of deficient of specific markers that discriminate within the subsets of immune cells, causing a break in our knowledge that how these subpopulations of immune cells act among normal and disease conditions. However, one strategic plan to determine new immune regulatory targets for regeneration therapeutics is to determine the specific markers and function of tissue-resident immune cells under homeostasis and injury. Although different phenotypes of pro/anti-inflammatory macrophages are extensively studied in the context of wound healing, fibrosis, and regeneration, other phenotypic subpopulations, for instance, nuetrophils and T-cells, need to be investigated to know their precise roles in tissue regeneration. Modulation of macrophage polarization seems to be successful in application. Thus, the current knowledge on the metabolic profiles of distinctly activated macrophages could be a promising target for therapeutic applications. Although metabolic molecular aspects of macrophages are still at the back, the metabolic switches are much more crucial.

There is little knowledge on the role of the adaptive immune system particularly T-cells in tissue regeneration. Does adaptive immunity facilitate tissue regeneration depending on antigen specificity? If so, how the T-cells are assigned and stimulated and function to counter self-antigens during injury, which are distinctive from reacting nonself antigens? At present, several therapies are hampered because of the lack of specific markers that identify different subpopulations of immune cells. T-regs in skeletal muscle, adipose, and colonic lamina propria are known to maintain tissue homeostasis [299]. It has been reported that IL-33 expands T-regs in the lamina propria, which facilitate local tissue repair with stimulation of amphiregulin. The precise role of T-regs in intestinal regeneration needs further examination.

The T-regs' suppressive actions play a role in supporting tissue repair. However, the allied contributions of skin-resident T-regs versus lymph nodes to suppress severe inflammation still need to be addressed. There is still far more to be discovered related to the metabolic reprogramming of immune cells because a diverse range of switches is involved in the phenotype transition. For example, L-PFK2 to the more active u-PFK2 during classical activation causes accumulation of fructose-2,6-bisphosphaste that drives glycolysis and simultaneous downregulation of sedoheptulose-7-phosphate.

Although citrate connects various critical pathways including carbohydrate, fatty acid, and protein transitions, its key role in generating acetyl-CoA that is involved in the acetylation of histones in modulating immune cell functions needs further investigation. Several studies discussed here have provided new mechanistic insight into how the delivery of immune signals can program tolerance and how material characteristics impact immune cell functions in disease settings.

Furthermore, the chronic inflammation sustains tissue remodeling and dysfunction in many diseases. Although the immune modulation by biomaterials has great capacity in therapy of several diseases, coordinating control over immune activities is urgently required. The majority of recent findings about the interactions of biomaterials including scaffolds, nanoparticles, and hydrogels with immune cells and stem cells suggest that appropriate design of biomaterials may modulate immune signality, which in turn affects regenerative capacity of stem cells and inflammation resolution in a complex microenvironment. Several studies have pointed out the initial immune response towards implanted biomaterials, but detailed strategies would be necessary to achieve optimal functional incorporation. The future biomaterials having structural and functional properties that resemble antigen self-assembling peptides may modulate adaptive immune response and thereby achieve appealing regeneration results. Failure to provide itemized understanding of biomaterial-immune cell interactions governing consequential pathological alterations in the microenvironment is a central barrier to the development of potential biomaterial-based therapies. Emerging strategies and solutions to prevent or decrease undesirable side effects in the usage of biomedical devices/implants still designate a major challenge in the tissue engineering field. Moreover, biomaterials with the integration of stem cells/bioactive elements provide an excellent scope to design novel therapies especially for inflammatory diseases of CNS. Finally, in the future, biomaterials can be used in emerging areas that illuminate immune tolerance. These include immune metabolisms, wherein exciting connections between immune functions and metabolisms are being discovered.

## Figures and Tables

**Table 1:** Metabolic pathways in different types of immune cells.

Type of immune cells	Metabolism^∗^	References
M1 macrophages	Aerobic glycolysis, PPP	[90, 91]
M2 macrophages	TCA, OXYPHOS, FA oxidation, amino acid	[11, 90, 92–94]
Neutrophils	Aerobic glycolysis, PPP	[95, 96]
Dendritic cells	Aerobic glycolysis, PPP	[97, 98]
Activated T-cells	Aerobic glycolysis	[99]
Regulatory T-cells (T-regs)	Fatty acid oxidation	[100]
T-memory cells	Fatty acid oxidation	[101]

^∗^TCA: tricarboxylic acid; OXYPHOS: oxidative phosphorylation; PPP: pentose phosphate pathway.

## Abbreviations

2-DG: 2-Deoxyglucose
2-OGDD: 2-Oxoglutarate-dependent dioxygenase
A2R: A2 adenosine receptor
ACLY: ATP-citrate lyase
ACO2: Aconitase 2
ADSCs: Adipose-derived mesenchymal stem cells
ALI: Acute lung injury
APC: Antigen-presenting cells
ARDS: Acute respiratory syndrome
Arg-1: Arginase-1
ATF3: Activating transcription factor 3
ATP: Adenosine triphosphate
bFGF: Basic fibroblast growth factor
BMDMs: Bone marrow-derived macrophages
BMSCs: Bone marrow-derived mesenchymal stem cells
CABG: Coronary artery bypass grafting
CARKL: Carbohydrate kinase-like
CAS: Carotid artery stenting
CCl_4_: Carbon tetrachloride
CD-73: Cluster of differentiation 73
CEA: Carotid endarterectomy
CIC: Citrate carrier
CNS: Central nervous system
COX2/COX1: Cyclooxygenase 2/1
DAMPs: Damage-associated molecular patterns
DDS: Drug-delivered systems
DEL-1: Developmental endothelial locus-1
DFU: Diabetic foot ulcer
DMI: Dimethyl itaconate
DMOG: Dimethyloxalylglycine
ECM: Extracellular matrix
ECs: Endothelial cells
ER: Endoplasmic reticulum
ERR*α*: Estrogen-related receptor-*α*
ETC: Electron transport chain
FA: Folic acid
FAK: Focal adhesion kinase
FAO: Fatty acid oxidation
FAS: Fatty acid synthase
FOXP3: Forkhead box P3
FTIAN: Fibrin-targeted imaging and antithrombotic nanomedicine
FA-TLR7-54: Folate-targeted TLR7 agonist
GABA: *γ*-Aminobutyric acid
GAPDH: Glyceraldehyde 3-phosphate dehydrogenase
GelMA: Gelatin methacryloyl
GlcNAc: Glutamine-UDPN- acetylglucosamine
GLUT1: Glucose transporter 1
GO: Graphene oxide
GPCR: G protein-coupled receptor
GSH: Glutathione
GST: Glutathione-S-transferase
HA: Hyaluronic acid
HIF-1*α*: Hypoxia-inducible factor 1-alpha
IBD: Inflammatory bowel disease
ICAM-1: Intercellular adhesion molecular-1
IDO: Indoleamine 2,3-dioxygenase
IGF-1: Insulin-like growth factor 1
IGF-1C: C domain of IGF-1 peptide
IKK*β*: Inhibitor of nuclear factor kappa-B kinase
IL: Interleukin
IL-4 pDNA: IL-4 plasmid DNA
INF-*γ*: Interferon gamma
iNOS: Inducible nitric oxide synthase
iPSC: Induced pluripotent stem cells
*IRG1*: Immune-responsive gene 1
IRPs: Iron regulatory proteins
I*κ*B*ζ*: Inhibitor of kappa B
Jmjd3: Jumonji domain-containing histone demethylase 3
LDL: Low-density lipoprotein
LDs: Lipid droplets
Lira: Liraglutide
LPS: Lipopolysaccharide
LXR: Liver X receptor
Ly6C: Lymphocyte antigen 6 complex
MAPK: Mitogen-activated protein kinase
MGL-1: Macrophage galactose-type C-type lectin 1
MHC: Major histocompatibility complex
MI: Myocardial infarction
MMPs: Metalloproteinases
MP: Methylprednisolone
mPGE2S: Microsomal prostaglandin E synthase
MPO: Myeloperoxidase
Mrc1: C-type mannose receptor 1
mRNA: Messenger RNA
MSCs: Mesenchymal stem cells
mTORC1: Mammalian target of rapamycin 1
MXF: Moxifloxacin
NADPH: Nicotinamide adenine dinucleotide phosphate
nCeO_2_: Cerium oxide nanoparticles
NETs: Neutrophil extracellular traps
NF-*κ*B: Nuclear factor kappa-light-chain-enhancer of activated B
NK: Natural killer
NLR: NOD-like receptor
NLRP3: NLR family pyrin domain containing 3
NO: Nitric oxide
NPC: Neural progenitor cells
NRF-1: Nuclear respiratory factor 1
Nrf2: Nuclear factor erythroid 2-related factor 2
NSAIDS: Nonsteroidal anti-inflammatory drugs
OXPHOS: Oxidative phosphorylation
PAMPs: Pathogen-associated molecular patterns
PCI: Percutaneous coronary intervention
PCSs: Porous collagen-based scaffolds
PDH: Pyruvate dehydrogenase
PDK1: Pyruvate dehydrogenase kinase 1
PD-L1: Programmed death-ligand 1
PEG-GelMa: Polyethylene glycol-gelatin-methacrylate
PGC-1*β*: PPAR*γ*-coactivator-1*β*
PGE2: Prostaglandin E2
PHBV: Poly(3-hydroxybutyrate-co-3-hydroxyvalerate)
PHD2: Prolyl-hydroxylase 2
PKM2: Pyruvate kinase isoenzyme M2
PKN: Protein kinase N
PLGA/Gel: Poly(lactide-co-glycolide)/gelatin
PPADT: Poly(1,4-phenleneacetonedimethylene thioketal)
PPAR-*γ*: Peroxisome proliferator-activated receptor gamma
PPP: Pentose phosphate pathway
PUTK: Polyurethane containing thioketal
rAAV: Recombinant adeno-associated virus
Reg3*β*: Regenerative islet-derived 3*β*
RET: Reverse electron transport
ROS: Reactive oxygen species
SAP: Self-assembling peptide
SAP: Serum amyloid P
SDH: Succinate dehydrogenase
siRNA: Small interfering RNA
SMCs: Smooth muscle cells
SOX9: SRY-box transcription actor 9
Src: Family kinases
STAT6: Signal transducer and activator of transcription 6
TAMs: Tumor-associated macrophages
TCA: Tricarboxylic acid
TET: T5-methylcytosine hydroxylases
TGF-*β*: Transforming growth factor-*β*
Th1/Th2: T helper cell type 1/2
TLR: Toll-like receptors
TNF-*α*: Tumor necrosis factor alpha
Tpl: Tempol
T-regs: Regulatory T-cells
Trx1: Thioredoxin 1
TXNDC5: Thioredoxin domain 5
UDP-GlcNAc: Uridine 5′-diphospho-N-acetylglucosamine
uPFK2: Ubiquitous phosphofructokinase-2
VCAM-1: Vascular cell adhesion protein 1
VEGF: Vascular endothelial growth factor
VLA-4: Very late antigen-4
vWF: von Willebrand factor
WBCs: White blood cells
*α*-KG: Alpha-keto glutarate
*α*-SMA: *α*-Smooth muscle actin.
